# Increasing NADPH impairs fungal H_2_O_2_ resistance by perturbing transcriptional regulation of peroxiredoxin

**DOI:** 10.1186/s40643-021-00489-w

**Published:** 2022-01-03

**Authors:** Jingyi Li, Yanwei Sun, Feiyun Liu, Yao Zhou, Yunfeng Yan, Zhemin Zhou, Ping Wang, Shengmin Zhou

**Affiliations:** 1 State Key Laboratory of Bioreactor Engineering, School of Biotechnology, East China University of Science and Technology, Shanghai, 200237 China; 2 Key Laboratory of Industrial Biotechnology (Ministry of Education), School of Biotechnology, Jiangnan University, 1800 Lihu Avenue, Wuxi, 214122 Jiangsu China; 3 Department of Bioproducts and Biosystems Engineering, University of Minnesota, Twin cities, Saint Paul, MN 55108 USA

**Keywords:** Peroxiredoxin, NADPH, Oxidative stress, *Aspergillus*, Glucose-6-phosphate dehydrogenase, AnCF

## Abstract

NADPH provides the reducing power for decomposition of reactive oxygen species (ROS), making it an indispensable part during ROS defense. It remains uncertain, however, if living cells respond to the ROS challenge with an elevated intracellular NADPH level or a more complex NADPH-mediated manner. Herein, we employed a model fungus *Aspergillus nidulans* to probe this issue. A conditional expression of glucose-6-phosphate dehydrogenase (G6PD)-strain was constructed to manipulate intracellular NADPH levels. As expected, turning down the cellular NADPH concentration drastically lowered the ROS response of the strain; it was interesting to note that increasing NADPH levels also impaired fungal H_2_O_2_ resistance. Further analysis showed that excess NADPH promoted the assembly of the CCAAT-binding factor AnCF, which in turn suppressed NapA, a transcriptional activator of PrxA (the key NADPH-dependent ROS scavenger), leading to low antioxidant ability. In natural cell response to oxidative stress, we noticed that the intracellular NADPH level fluctuated “down then up” in the presence of H_2_O_2_. This might be the result of a co-action of the PrxA-dependent NADPH consumption and NADPH-dependent feedback of G6PD. The fluctuation of NADPH is well correlated to the formation of AnCF assembly and expression of NapA, thus modulating the ROS defense. Our research elucidated how *A. nidulans* precisely controls NADPH levels for ROS defense.

**Graphical Abstract:**

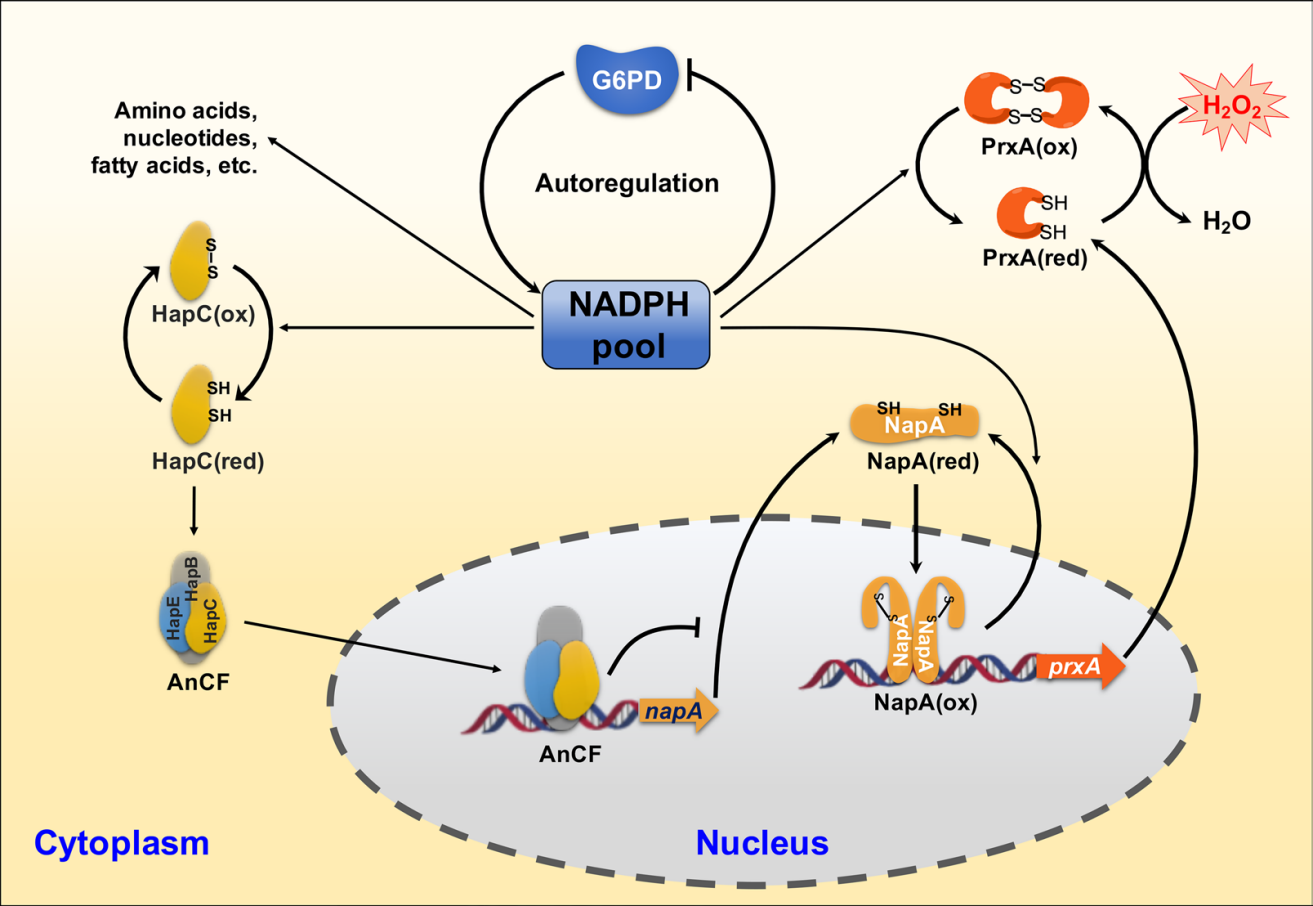

**Supplementary Information:**

The online version contains supplementary material available at 10.1186/s40643-021-00489-w.

## Introduction

Reactive oxygen species (ROS) are produced in a wide range of physiological processes, including aerobic respiration (Kalyanaraman et al. 2018), exposure to environmental agents such as UV irradiation (Kuehne et al. 2015) and drugs/xenobiotics (Van Acker et al. 2017), and immune response in higher organisms (Nathan et al. 2013; Yang et al. 2013). The resultant ROS can lead to oxidative stress by directly or indirectly damaging DNA, proteins, and lipids (Nathan et al. 2013). Cells have evolved a variety of enzymes, such as glutathione reductase, superoxide dismutase, and peroxidases, to defend against ubiquitous ROS toxicity. Among such defense mechanisms, the thioredoxin peroxidase enzymes, peroxiredoxins (Prx), are the most abundant antioxidants and is widespread among archaea, bacteria, and eukaryotes (Poole et al. 2011; Rhee 2016). Prx are highly reactive with H_2_O_2_, using the reversible oxidation of cysteine residues to reduce peroxides. The resulting Prx disulfides are reduced by thioredoxin (Trx) using electrons from NADPH in the presence of Trx reductase (TrxR). We have previously shown that *Aspergillus nidulans* Prx (PrxA) is not essential to sustain normal growth, but lack of PrxA results in hypersensitivity of strains toward oxidative stress, indicating a specific biological function for exogenous ROS detoxification (Xia et al. 2018). A similar result was also obtained with the Prx ortholog in *A. fumigatus* (Hillmann et al. 2016; Rocha et al. 2018), suggesting PrxA has a key role in H_2_O_2_ resistance in *Aspergillus* species.

The CCAAT-binding complex is an important general transcriptional regulator for that transcription of numerous genes, including prosurvival and cell cycle-promoting genes (Oldfield et al. 2014; Hortschansky et al. 2017). The corresponding *A*. *nidulans* CCAAT-binding factor (AnCF) consists of the subunits HapB, HapC, and HapE (Thon et al. 2010). AnCF is regulated at the posttranscriptional level by the redox status of the cell, thereby serving as a redox sensor coordinating the cellular oxidative stress response. AnCF senses the cellular redox status via oxidative modification of thiol groups in HapC. Oxidized HapC is unable to participate in AnCF assembly, leading to abolishment of the regulation role, while, the invalid HapC can be revived via the reduction by Trx system (Thon et al. 2010). AnCF acts as a repressor of *napA*, which then encodes the ROS-specific transcriptional activator NapA whose target genes include *A. nidulans* Trx-encoding gene (*trxA*), catalase-encoding gene (*catB*), and PrxA-encoding gene (*prxA*) (Thon et al. 2010). Therefore, ROS deactivates AnCF and then increases expression of *napA* via release of AnCF repression for activation of oxidative defense mechanisms.

NADPH serves as an important reducing equivalent and is essential in cellular defense against oxidative damage. In many biological systems, the antioxidant functions of NADPH are exerted via regeneration of Trx by TrxR. NADPH-reduced Trx may provide reducing equivalents to Prx, as well the other proteins containing oxidized cysteine groups, including NapA and HapC, through a thiol–disulfide interaction (Thon et al. 2010). To replenish exhausted inventories of intracellular NADPH, numerous pathways are known to be involved in NADPH regeneration (Wang et al. 2014; Zhou et al. 2016). Among them, the pentose phosphate pathway (PPP) is believed to be the major one, in which NADPH is produced by two enzymes, namely glucose-6-phosphate dehydrogenase (G6PD), 6-phosphogluconate dehydrogenase (6PGD). Although NADPH is considered an indispensable reducing agent for ROS elimination, how NADPH manipulates cellular ROS resistance remains obscure, and many contradictory results can be gleaned from previous reports. For example, animal cell studies demonstrated that increases in NADPH levels by overexpressing of the corresponding enzymes could protect cells or tissues against oxidative damage (Xiao et al. 2020). The advantages brought by excess production of NADP(H) are due to its functional roles as an indispensable cofactor for glutathione reductase (GR) and TrxR that are essential for GPx- and Prx-mediated peroxide removal, respectively. However, many other studies have supported the opposite notion that excess levels of intracellular NADPH can induce reductive stress and cellular dysfunction. This opposite conclusion was drawn from the fact that excess NAD(P)H may be used by NADPH oxidases (NOXs) to produce ROS (Brandes et al. 2014; Yu et al. 2014; Xiao et al. 2020). Therefore, NAD(P)H serves as dual-function participants, either an antioxidant cofactor or a pro-oxidant, to maintain cellular redox homeostasis in many animal cells. Notably, the effect of excess NADPH on microbes is yet to be investigated, though overexpression of NADPH-producing enzymes has been regarded as a promising strategy in NADPH-required metabolic engineering (Xue et al. 2017).

Here, we used the filamentous fungus *A. nidulans*, a classical model of pathogenic and commercial fermentation *Aspergillus* species, to investigate the relationship between NADPH generation and fungal ROS defense. Our findings indicated that although NADPH is an indispensable reducing agent for ROS elimination, increasing NADPH levels by modulating the expression strength of typical NADPH-generating enzymes produced an adverse effect on fungal resistance to H_2_O_2_. We found that excess NADPH promoted the assembly of AnCF, which in turn suppressed NapA, leading to low levels of PrxA and the eventual impairment of antioxidant ability. Our results provide new insights into the dual-function role of NADPH in maintaining cellular redox homeostasis.

## Materials and methods

### Strains and growth conditions

*Aspergillus nidulans* is the experimental model used in this study. Genotypes of strain used in this study are listed in Additional file 1: Table S1. All fungi were grown at 37 °C in minimal medium (MM) (1% glucose, 10 mM NaNO_3_, 10 mM KH_2_PO_4_, 7 mM KCl, 2 mM MgSO_4_, 2 mL L^−1^ Hunter’s trace metals, pH 6.5) (Kadooka et al. 2016), supplemented appropriately (0.4 mg/L biotin, 0.5 g/L uracil, 0.6 g/L uridine, 0.4 mg/L pyridoxine). *E. coli* DH5α and BL21 (DE3) were used for gene cloning and protein expression, respectively. Sodium nitrate (10 mM), proline (10 mM) and ammonium tartrate (5 mM) were used as sole nitrogen sources for *niaD* promoter replacement strains according to the experimental requirement.

### Construction of gene disruption strains

Primers are listed in Additional file 1: Table S2. *A. nidulans* ABPUN genomic DNA was obtained using Wizard Genomic DNA Purification Kit (Promega, USA), and used as template to produce the gene deletion constructs. All PCR was performed using PrimeSTAR HS DNA Polymerase (Takara, Japan). CRISPRdirect (crispr.dbcls.jp) was used for sgRNA protospacer selection. The sgRNAs were synthesized using *A. nidulans* ABPUN genomic DNA; primers pairs *AN4034*-sgF1/*AN4034*-sgR1 (for Δ*hapC*), *AN2981*-sgF1/*AN2981*-sgR1 (for Δ*AN2981*), *AN2981*-sgF2/*AN2981*-sgR2 (for Δ*AN2981*), *AN3954*-sgF1/*AN3954*-sgR1 (for Δ*AN3954*), *AN3954*-sgF2/*AN3954*-sgR2 (for Δ*AN3954*) were prepared using a GeneArt^TM^ Precision gRNA Synthesis Kit (Invitrogen, USA) in accordance with the manufacturer’s instructions. In vitro cleavage activity tests were performed using the PC1400 Kit (Inovogen Tech. Co., China). The *pyrG* marker gene was amplified using *A. oryzae* RIB40 genomic DNA and the primers, *pyrG*-F1 and *pyrG*-R primers. The marker gene *argB* was amplified using *A. nidulans* A6 genomic DNA and the primers, *argB*-F and *argB*-R. To delete *hapC* (*AN4034*) gene, primer pairs *AN4034*-uF/*AN4034*-uR and *AN4034*-dF/*AN4034*-dR were used to amplify the 1 kb of the 5′ and 3′ untranslated regions (UTR) of *AN4034*, respectively. Primer *AN4034*-nested-F and *AN4034*-nested-R were used to amplify the final fusion product. The resultant DNA cassette 5′*AN4034*-*argB*-3′*AN4034* (400 ng), together with the corresponding sgRNA (100 nM) and 1 μg purified Cas9 (Inovogen Tech. Co.), was introduced into *A. nidulans* ABPUN strain as previously described method (Kitamoto 2002; Pohl et al. 2016) to create an *hapC* deletion strain *∆hapC*. Disruptions of the *gsdA* (*AN2981*) gene were performed using the same methods except that the marker gene *pyrG* was employed for *gsdA* disruption. Transformants were selected from the plates based on their auxotrophy and confirmed by colony PCR (Additional file 1: Fig. S1) using KOD FX polymerase (Toyobo, Japan). The genotype of the resultant disruptants is shown in Additional file 1: Fig. S1.

### Construction of promoter substitution strains

The corresponding primers are listed in Additional file 1: Table S2. *nP*.*gsdA* was constructed as follows. *niaD* promoter (*niaD*.P) was cloned from *A. nidulans* ABPUN genomic DNA using the primers *niaD-*F and *niaD*-R. Marker gene *pyrG* from *A. oryzae* was amplified using *A. oryzae* RIB40 genomic DNA with primers *pyrG*-F2 and *pyrG*-R. The resultant *niaD*.P was fused with *pyrG* was to generate *pyrG*-*niaD.*P fragment by an overlapping PCR using the two primers *pyrG*-F1 and *niaD*-nested-R. Approximately 1 kb of 5′ UTR and 1 kb of *gsdA* open reading frame (ORF) were cloned from *A. nidulans* ABPUN genomic DNA using primer pairs *AN2981*-5′-F/*AN2981*-5′-R and *AN2981*-3′-F/*AN2981*-3′-R, respectively. To construct the *pyrG*-*niaD*.P-*gsdA* cassette, *pyrG*-*niaD*.P was flanked with the resulted 5′ UTR and ORF of *gsdA* by fusion PCR using the *AN2981*-nested-F and *AN2981*-nested-R primers. The resultant PCR product was transformed to ABPUN strain to obtain *nP*.*gsdA* strain. The strains, *gP*.*gsdA* and *gP*.*gndA* were constructed using the same strategy with their corresponding primers (Additional file 1: Table S2) and shown in Additional file 1: Fig. S2.

A recombinant pUC19-*pyroA*-*gpdA*.P-*prxA*-T*trpC* plasmid was constructed to obtain the *gP*.*prxA* strain. In this plasmid, the *gpdA* promoter (*gpdA*.P) was cloned from *A. nidulans* ABPUN genomic DNA using primers *gpdA*-F and *gpdA*-R and then fused to the ORF of *A. nidulans prxA* and *E. coli* terminator T*trpC* with primer pair pUC19-*pyroA*-*gPprxA*-F/pUC19-*pyroA*-*gPprxA*-R. Then, the fused fragment was inserted into pUC19-*pyroA* plasmid (our lab) using the ClonExpress II One Step Cloning Kit (Vazyme, China). The resultant *gP*.*prxA* strain was further transformed with *pyrG*-*niaD*.P-*gsdA* cassette to construct *nP*.*gsdA*/*gP*.*prxA* strain. The *pyrG*-*niaD*.P-*gsdA* cassette was introduced into Δ*hapC* strains to generate the strain *nP*.*gsdA*/Δ*hapC*. The successful disruptants were confirmed using colony PCR with the corresponding primers indicated in Additional file 1: Fig. S2.

### Construction of GFP-tagged NapA, GFP-tagged PrxA and Flag-tagged HapC expression strains

Primers are listed in Additional file 1: Table S1. The cassette for expressing C-terminal tagged GFP of NapA was constructed as follows. The marker gene *pyroA* was amplified by PCR using *A. nidulans* A6 genomic DNA with primers *pyroA*-F and *pyroA*-R. The gene for expressing GFP with an N-terminal 5GA linker was cloned from pUC19-*gfp* plasmid (our lab) with primers *gfp*-F and *gfp*-R. An overlapping PCR was performed to obtain the *gfp*::*pyroA* fragment with the primers, *gfp*-*pyroA*-F and *gfp*-*pyroA*-R. Approximately 2.8 kb of 5′ UTR plus the ORF and 1 kb of 3′ UTR of *napA* were cloned from *A. nidulans* A6 genomic DNA with the corresponding primer pairs *napA*-5′-F/*napA*-5′-R and *napA*-3′ UTR-F/*napA-*3′ UTR-R. The cassette (*napA*-*gfp*-3′*napA*) containing *gfp*::*pyroA* flanked with 5′ UTR plus the ORF and 3′ UTR of *napA* was constructed by fusion PCR using the above-mentioned three resultant PCR products with the nested primers *napA-*5′-nested-F/*napA-*3′ UTR-nested-R. The resultant cassette was transformed into the strain WT_*argB* (our lab) to construct GFP-tagged NapA-expression strain (N_Gfp) as shown in Additional file 1: Fig. S3. One of the successful transformants was verified using colony PCR and sequenced at Tsingke Biotechnology Co.. The resultant N_Gfp strain was further introduced with *pyrG*-*niaD*.P-*gsdA* cassette to obtain the strain *nP*.*gsdA*/N_Gfp.

For the N-terminal tagged GFP of PrxA expression strain, a pUC19-*pyroA*-*gfp*-*prxA* plasmid was first constructed. Approximately 1 kb of 5′ UTR and 1.6 kb of 3′ UTR plus ORF of *prxA* was cloned from *A. nidulans* A6 genomic DNA with the corresponding primer pairs *prxA*-uF/*prxA*-uR and *prxA*-dF/*prxA*-dR. The gene expressing GFP with a C-terminal 5GA linker was cloned from a pUC19-*gfp* plasmid (our lab) with primers *gfp*-cF and *gfp*-cR and then fused with the two PCR products with the nested primers pUC19-*prxA-*nested-F/pUC19-*prxA*-nested-R. Next, the pUC19-*pyroA* plasmid was digested by *Sma* I and ligated with the above fusion PCR product using a ClonExpress II One-Step Cloning Kit (Vazyme). The resulting plasmid pUC19-*pyroA*- *gfp*-*prxA* was introduced into the Δ*prxA* strain to generate P_Gfp. One of the successful transformants was verified using colony PCR, as indicated in Additional file 1: Fig. S3. The *pyrG*-*niaD*.P-*gsdA* cassette was also introduced into P_Gfp to obtain the strain *nP*.*gsdA*/P_Gfp.

For the Flag-tagged HapC expression strain, a pUC19-*pyroA*-*hapC*-Flag plasmid was first constructed. A DNA fragment containing approximately 1.5 kb 5′ UTR followed by HapC-Flag-encoding DNA was amplified using *A. nidulans* ABPUN genomic DNA as template with the primers *hapC*-uF and *hapC*-Flag-R. Approximately 1 kb of 3′ UTR of *hapC* was cloned using the same template with primer pairs Flag-*hapC*-F and *hapC*-dR. The two resultant PCR products were fused by overlapping PCR with the primers pUC19-*pyroA*-*hapC*-uF and pUC19-*pyroA*-*hapC*-dR. Plasmid pUC19-*pyroA* was digested by *Hind* III and *Sma* I and ligated with the above fusion PCR product using a ClonExpress II One Step Cloning Kit (Vazyme). The resulting plasmid pUC19-*pyroA*-*hapC*-Flag was introduced into Δ*hapC* and *nP*.*gsdA*/Δ*hapC* strain to generate H_Flag and *nP*.*gsdA*/H_Flag strains, respectively. One of the successful transformants was verified using colony PCR as shown in Additional file 1: Fig. S4, and the resultant PCR products were sequenced for further confirmation at Tsingke Biotechnology Co.

### Recombinant AnG6PD preparation

The DNA encoding AnG6PD was amplified from *A. nidulans* cDNA using the primers pET28a-*gsdA*-F/pET28a-*gsdA*-R (Additional file 1: Table S2). The DNA fragment was inserted between *Nde* I and *Xho* I restriction sites of pET28a (+) vector. *E. coli* BL21 (DE3) cells transformed with expression plasmid was cultured in LB medium supplied with 50 μg/mL kanamycin. The protein expression was induced at 30 °C with 0.2 mM isopropyl-β-d-thiogalactoside (IPTG). The recombinant protein was purified by affinity chromatography with HisTrap FF column (GE Healthcare, USA) and confirmed using SDS-PAGE analysis (Additional file 1: Fig. S5).

### Sensitivity of *A. nidulans* to H_2_O_2_

Serial dilutions of 48-h cultivated *A. nidulans* conidia were spotted onto MM plates containing indicated concentrations of H_2_O_2_, and then incubation at 37 °C for 2 days. The morphology of the colonies was examined to determine their sensitivity to H_2_O_2_. For the conidia survival assay, conidia (1 × 10^3^ mL^−1^, 10 μL) were suspended in MM containing top agar (0.75% agar), and the indicted concentrations of H_2_O_2_ were then spread on MM plates containing the same concentrations of H_2_O_2_. Colonies were counted after a 48-h incubation, and CFU were expressed as percentages of the CFU for strains incubated without H_2_O_2_.

### G6PD activity assay

Intracellular G6PD enzyme activity was determined in cell lysates by measuring the rate of increase of NADPH at 340 nm (UV-5100 Spectrophotometer, Hitachi, Japan). The assay was performed at 25 °C in 1 mL containing 100 mM Tris–HCl buffer (pH 8.0), 20 mM MgCl_2_, 5 mM D-glucose-6-phosphate, 0.3 mM NADP^+^, and 50 mg cell lysates. G6PD activity was expressed in U/mg protein; 1 unit (1 U) of G6PD was defined as 1 mg/mL of enzyme required to produce 1 µmol NADPH in 1 min at 25 °C.

### Fluorescence microscopy imaging of NapA-GFP strains

Approximately 10^5^ conidia were suspended in 200 µL MM medium, seeded in a 35-mm confocal dish, and incubated at 37 °C for 10 h. Samples were treated with or without 2 mM H_2_O_2_ and incubated for 20 min, and then the fluorescent mycelia were imaged by laser confocal microscope (TCS SP8, Leica, Germany). Nuclei were stained with Hoechst 33258 for 15 min, and then washed by PBS for three times before the observation.

### Native and denaturing PAGE and western blot analysis

Strains expressing Flag-tagged HapC (H_Flag, *nP*.*gsdA*/H_Flag) were precultured in MM medium with nitrate as sole nitrogen source for 16 h, and then incubated with 1 mM H_2_O_2_ at 37 °C for 30, 90, and 120 min. Protein was extracted as described (Thon et al. 2010), using a non-denaturing procedure in lysis buffer (100 mM pH 8.0 Tris–HCl, 1 mM EDTA and 1% 1 × protease inhibitor mixture). For denaturing PAGE analysis, protein was extracted using lysis buffer supplied with 1% SDS. Protein concentrations of the cell tracts were measured by Bradford assay (Sangon), and then samples were diluted to a protein concentration of 1 mg/mL. Native PAGE was performed using a running buffer of 25 mM Tris–HCl (pH 8.0) and 195 mM glycine. Samples (100 µg per lane) were run for 2 h using a commercial 4–20% gradient gels (Beyotime) and then analyzed by western blotting with a PVDF membrane. For denaturing conditions, SDS-PAGE was performed using SDS running buffer containing 0.1% SDS and 12% SDS-PAGE gels. The AnCF-Flag was detected using an anti-Flag antibody (Transgen). The secondary antibody for Flag was anti-mouse IgG HRP conjugate (Transgen). The blots were subsequently reprobed for Actin using an anti-Actin antibody (Sigma-Aldrich) and anti-rabbit IgG HRP conjugate (Transgen, China). The ECL detection system (Tanon) was used to visualize proteins. Quantitative densitometric analyses of western blots were conducted using ImageJ.

### Quantification real-time PCR analysis

Total RNA was isolated using EZ-10 DNAaway RNA Mini-Preps Kit (Sangon, China). cDNAs were then reverse-transcribed with ReverTra Ace qPCR RT Master Mix with gDNA Remover (Toyobo, Japan). Quantitative PCR was preformed using a SYBR Green PCR Kit (Toyobo) and conducted on a CFX-96 Real-Time PCR system (Bio-Rad, USA). Primer pairs q-RT-*prxA*-F/q-RT-*prxA*-R, q-RT-*gsdA*-F/q-RT-*gsdA*-R, q-RT-*gndA*-F/q-RT-*gndA*-R, q-RT-*napA*-F/q-RT-*napA*-R, and q-RT-*actA*-F/q-RT-*actA*-R (Additional file 1: Table S2) were designed to amplify *prxA*, *gsdA*, *gndA*, *napA*, and *actA*, respectively. Relative mRNA levels were normalized to reference gene *actA*.

### Measurement of NADPH/NADP^+^ ratio

Mycelium was collected and ground in liquid nitrogen and then resuspended in 300 μL extraction buffer as described in the instruction manual (Sigma-Aldrich, USA). Cell lysates were filtered using a 10-kDa Ultra filter (Millipore, Sigma) to minimize the influence of NADPH-consume proteins. The NADPH/NADP^+^ ratio was calculated as [NADPH/ (total NADPH–NADPH)].

### Quantification analyses of intracellular GFP

Strains expressing GFP-tagged Prx (P_Gfp, *nP*.*gsdA*/P_Gfp) were precultured in MM medium with nitrate as a sole nitrogen source for 16 h and then incubated with or without 1 mM H_2_O_2_ at 37 °C for 2 h. Cells were collected and then disrupted with liquid nitrogen. The fluorescence values of the supernatant of cell lysates were measured using a fluorescence spectrophotometer (F-4600, Hitachi, Japan) at an excitation wavelength of 488 nm and an emission wavelength of 509 nm.

### Quantification analyses of intracellular ROS

Cell-permeable BES-H_2_O_2_-Ac (Wako, Japan) and BES-So-AM (Wako, Japan) were used as H_2_O_2_^−^- and O_2_^·−^-specific fluorescent probes, respectively. Precultivated fungal cells (16 h) were incubated with individual probes for 30 min before exposing these cells to H_2_O_2_ (1 mM) for 30 min. The ROS scavenger N-acetyl-l-cysteine (NAC) (Sigma-Aldrich, USA) was added to block H_2_O_2_ generation, cells were pretreated with or without 10 mM NAC for 1 h at 37 °C prior to incubation with BES-H_2_O_2_-Ac probes. Mycelia were then washed thrice using PBS, immediately ground into powder with liquid nitrogen, and then suspended in 50 mM PBS. The supernatant of the disrupted mycelia was analyzed using a fluorescence spectrophotometer (F-4600, Hitachi, Japan) at an excitation wavelength of 485 nm and an emission wavelength of 515 nm for H_2_O_2_ detection and an excitation wavelength of 505 nm and an emission wavelength of 544 nm for O_2_^·−^ detection.

### Statistical analysis

All experiments were repeated at least three times on independently generated samples with similar results. Representative experiments or the quantitative densitometric analyses of several experiments are shown, data are represented as mean ± SD. *P* < 0.05 was considered significant.

## Results

### NADPH-consuming PrxA is essential to cell survival under H_2_O_2_ stress conditions

**Fig. 1:**
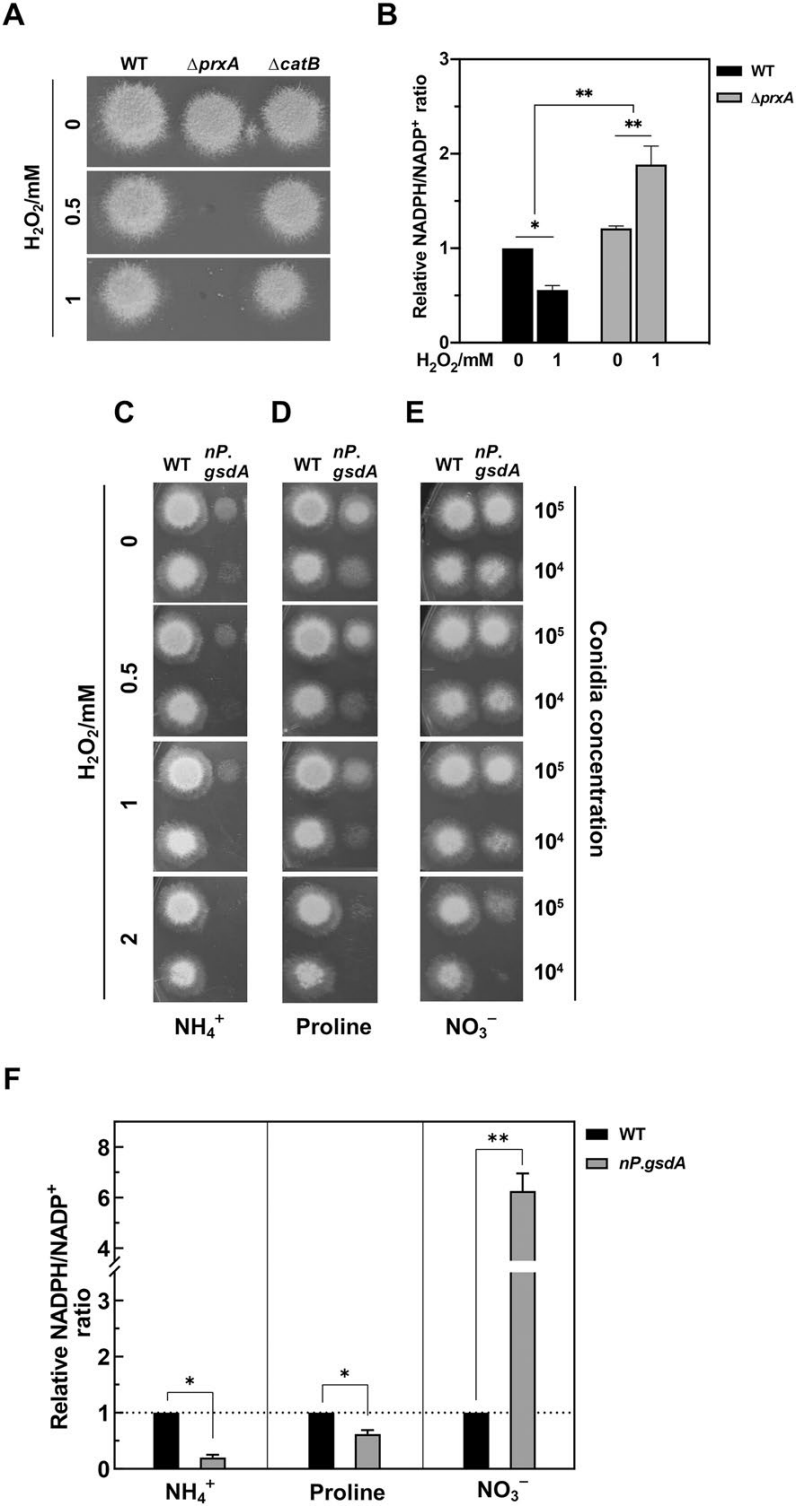
*A. nidulans* PrxA in tandem with G6PD is essential to fungal ROS defense. **A** Effects of disrupting *prxA* and *catB* on protection against H_2_O_2_. Fresh conidia (1 × 10^5^) of WT (WT_*argB*), ∆*prxA*, and ∆*catB* were inoculated on MM plates containing H_2_O_2_ at indicated concentrations and then incubated at 37 °C for 2 days. **B** Lack of PrxA resulted in intracellular accumulation of NADPH. After culture in liquid MM for 16 h, strains were treated with or without 1 mM H_2_O_2_ for 30 min before collection for determination of the NADPH/NADP^+^ ratio in cell lysates. NADPH/NADP^+^ is presented in relative quantitation; NADPH/NADP^+^ value of unstressed WT was set to 1 (mean ± SD; *n* = 3, *P < 0.05, **P < 0.01, one-way ANOVA). **C**–**E** Phenotypes of WT (WT_*pyrG*) and *nP*.*gsdA* strains on MM plates under H_2_O_2_ conditions using ammonium tartrate (left), proline (middle), or nitrate (right) as the sole nitrogen source. **F** NADPH/NADP^+^ ratios in WT (WT_*pyrG*) and *nP*.*gsdA* strains. Strains were cultivated in liquid MM with ammonium tartrate (left), proline (middle), or nitrate (right) as the sole nitrogen source for 16 h, and then the NADPH/NADP^+^ ratios in cell lysates were quantified and compared; NADPH/NADP^+^ value of WT cultivated with each nitrogen source was set to 1 (mean ± SD; *n* = 3, *P < 0.05, **P < 0.01, *t*-test)

We have previously shown that *A. nidulans* lacking PrxA displayed pronounced sensitivity to H_2_O_2_ (Xia et al. 2018), whereas, in many living beings, catalases act as the key H_2_O_2_-detoxifying enzyme (Rodriguez-Segade et al. 1985). Considering that catalases are abundant in *A. nidulans* (Kawasaki et al. 1997; Kawasaki et al. 2001), we directly compared the H_2_O_2_ protection functions exerted by PrxA and those of catalase B (a major catalase in *A. nidulans*) (Fig. 1A). Mutants carrying deletions in these genes (∆*prxA* or ∆*catB*) were viable with identical growth on agar plates to wild-type *A. nidulans* (WT) under normal growth conditions. Growth of ∆*prxA* was completely inhibited with 0.5 mM H_2_O_2_, whereas ∆*catB* exhibited little sensitivity to H_2_O_2_, clearly indicating that PrxA, rather than catalase B, is the indispensable enzyme that protects *A*. *nidulans* against H_2_O_2_ stress.

To confirm the process where PrxA employs NADPH to decompose H_2_O_2_ in vivo, we calculated the changes of intracellular NADPH/NADP^+^ ratio in WT and ∆*prxA* under oxidative stress conditions caused by H_2_O_2_. As expected, exposing WT to 1 mM H_2_O_2_ significantly decreased the NADPH/NADP^+^ ratio, which is considered to be the result of the NADPH being used for H_2_O_2_ decomposition (Fig. 1B). The ∆*prxA* strain was determined to have a slightly increased NADPH/NADP^+^ ratio under normal conditions in comparison with that of WT. In sharp contrast to the WT, H_2_O_2_ exposure further increased the NADPH/NADP^+^ ratio in ∆*prxA* (Fig. 1B), indicating that PrxA consumes NADPH to decompose H_2_O_2_. Taken together, we concluded that the NADPH-consuming PrxA plays an essential role in H_2_O_2_ detoxification.

### Decreasing NADPH impairs antioxidant ability

One of the major NADPH-producing enzymes in *A. nidulans* is identified as glucose-6-phosphate dehydrogenase (G6PD, encoded by *AN2981*) (Wennekes et al. 1993). To reveal the direct link between intracellular NADPH and cellular defense against oxidative stress in *A. nidulans*, we attempted to construct and phenotypically characterize the G6PD deficiency strain (∆*gsdA*). However, only heterokaryon mutants were obtained (data not shown), suggesting that *gsdA* may be essential for cell development and growth in *A. nidulans*. To analyze the functions of the potentially essential gene on oxidative stress resistance, we used the conditional promoter replacement strategy (Marchegiani et al. 2015). This strategy uses the *niaD* promoter (*niaD.*P), a nitrogen-regulated promoter from *A. nidulans*, to replace the endogenous promoter of a target gene to enable strict regulation. Up- and down-regulated expression can be achieved in the presence of NO_3_^−^ and NH_4_^+^ as the sole nitrogen source, respectively. Additionally, proline can be used as a neutral nitrogen source to partially derepress the activity of *niaD.*P from NH_4_^+^ suppression. Using this strategy, the conditional mutant *nP*.*gsdA* was successfully constructed (Additional file 1: Fig. S2).

In the absence of H_2_O_2_, *nP*.*gsdA* exhibited drastically attenuated growth under NH_4_^+^ repression conditions (Fig. 1C). The addition of proline partially relieved growth inhibition from NH_4_^+^ repression, whereas the addition of NO_3_^–^ almost recovered the growth rate compared with that of WT under unstressed conditions (Fig. 1D–E). These diverse phenotypes of the conditional mutant responding to the three nitrogen sources further supported the deduction that G6PD is important for fungal development. To provide insights into how intracellular NADPH levels affect the cell growth rate, we measured the intracellular NADPH/NADP^+^ ratios of *nP*.*gsdA* and found profound fluctuation of the NADPH/NADP^+^ ratio in response to different nitrogen sources (Fig. 1F). NO_3_^−^ induced a sixfold higher NADPH/NADP^+^ ratio in *nP*.*gsdA* than that in WT, whereas proline and NH_4_^+^ decreased the ratio to 2/3 and 1/5 of that of WT, respectively. Obviously, depressing of G6PD decreased intracellular NADPH, which should be responsible for fungal growth retardation.

Next, we investigated how NADPH decrease affects resistance ability of the fungus to oxidative stress. Although *nP*.*gsdA* remained alive under NH_4_^+^ conditions, the poor cellular growth should make it difficult to estimate the severity of the H_2_O_2_ damage under these conditions (Fig. 1C); therefore, we compared conidial viabilities in response to H_2_O_2_ treatment between WT and *nP*.*gsdA* strains by counting the colonies formed. The survival of *nP*.*gsdA* was poorer than that of WT under the oxidative stress conditions induced by 1 mM H_2_O_2_ (Additional file 1: Fig. S6A). Consistent with this result, the activity of G6PD and the corresponding NADPH/NADP^+^ ratio were significantly repressed by NH_4_^+^ (Additional file 1: Fig. S6B–C). Together with the fact that the slight derepression of *gsdA* by proline partially alleviated the H_2_O_2_ resistance defect of the mutant (Fig. 1D), we may conclude that the artificial down-regulation of NADPH levels impairs fungal H_2_O_2_ resistance ability. This is in agreement with the above-mentioned finding that the indispensable antioxidant PrxA employs NADPH for ROS elimination.

### Increasing NADPH also impairs cell antioxidant ability

**Fig. 2:**
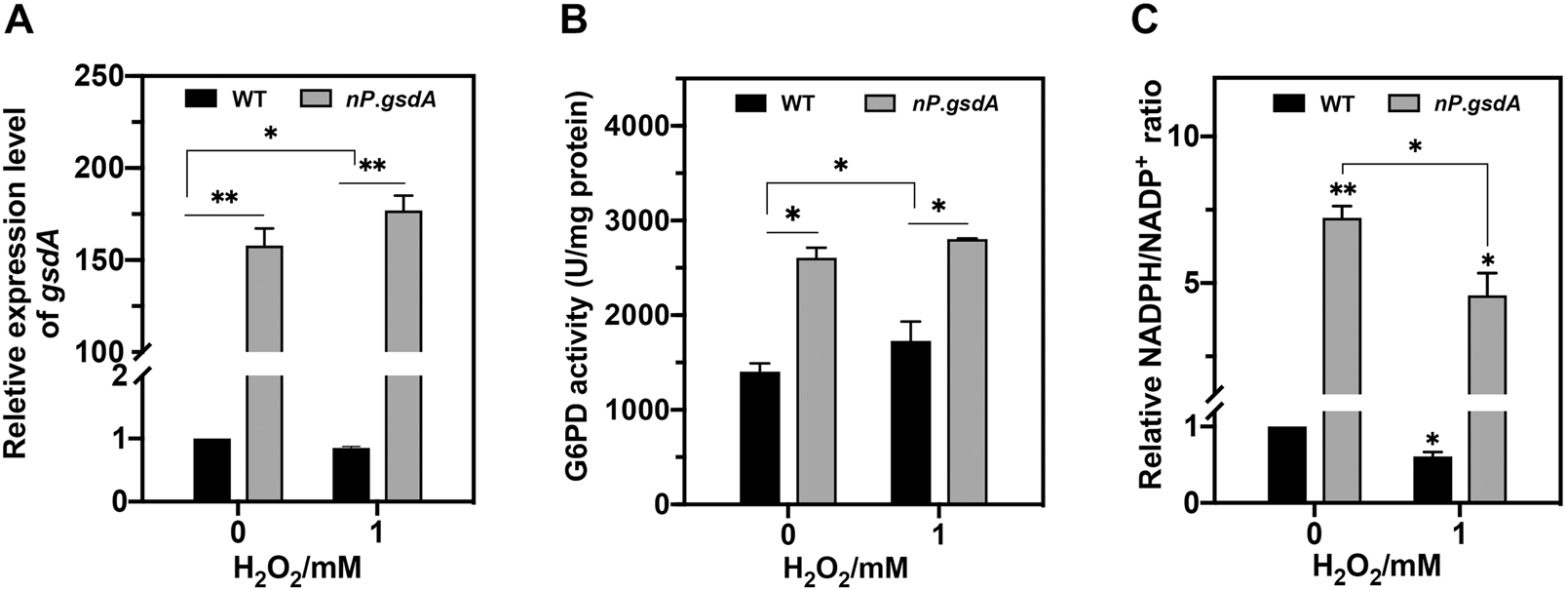
NO_3_^−^ induction promoted transcription (**A**), activity (**B**), NADPH production (**C**) of G6PD in *nP*.*gsdA* strains. **A**–**C** Fresh conidia (1 × 10^8^) of WT (WT_*pyrG*) and *nP*.*gsdA* strains were cultivated in MM medium using NO_3_^−^ as the nitrogen source for 16 h and then exposed to the indicated concentrations of H_2_O_2_ for 30 min for the following analysis (mean ± SD; *n* = 3, *P < 0.05, **P < 0.01, one-way ANOVA)

Given that the intracellular NADPH level is crucial for fungal antioxidant ability, increasing intracellular NADPH levels may be beneficial for fungal oxidative defense, as in *Drosophila melanogaster* and some other animal cells (Salvemini et al. 1999; Leopold et al. 2003; Legan et al. 2008; Zhang et al. 2012; Xiao et al. 2018, 2020). In our study, we found that NO_3_^−^ significantly induced *gsdA* expression (Fig. 2A) and accelerated G6PD activity (Fig. 2B), which resulted in at least a fivefold higher NADPH/NADP^+^ ratio in *nP*.*gsdA* than that in WT under either unstressed or stressed conditions (Fig. 2C). However, unexpectedly, *nP*.*gsdA* showed higher H_2_O_2_ sensitivity than that of WT (Fig. 1E), which led us to consider that increasing NADPH did not promote and, on the contrary, impaired cell antioxidant ability.

For further verification of this hypothesis, we constructed two other NADPH-high producing strains, *gP*.*gsdA* and *gP*.*gndA.* A constant and high-yield of NADPH was expected to be realized by replacing the native promoters of *gsdA* and *gndA* (6PGD encoding gene) with *gpdA* promoters, which is a strong constitutive promoter derived from the *A. nidulans gpdA* gene that encodes glyceraldehyde-3-phosphate dehydrogenase (Umemura et al. 2020). In *gP*.*gsdA*, the *gpdA* promoter produced approximately 100-fold more *gsdA* mRNA than that produced by the native *gsdA* promoter, but only half of that was produced by the *niaD* promoter (Additional file 1: Fig. S7A). The intracellular NADPH levels ranged from high to low across the *nP*.*gsdA*, *gP*.*gsdA*, and WT strains (Additional file 1: Fig. S7B), which was contrary to the orders of fungal H_2_O_2_ resistance (Additional file 1: Fig. S7C). In *gP*.*gndA*, both *gndA* mRNA and intracellular NADPH levels were significantly elevated by the *gpdA* promoter, which also lowered its antioxidant ability (Additional file 1: Fig. S8A–C). These results strengthened the fact that artificial increasing NADPH levels has adverse effects on fungal H_2_O_2_ resistance.

**Fig. 3:**
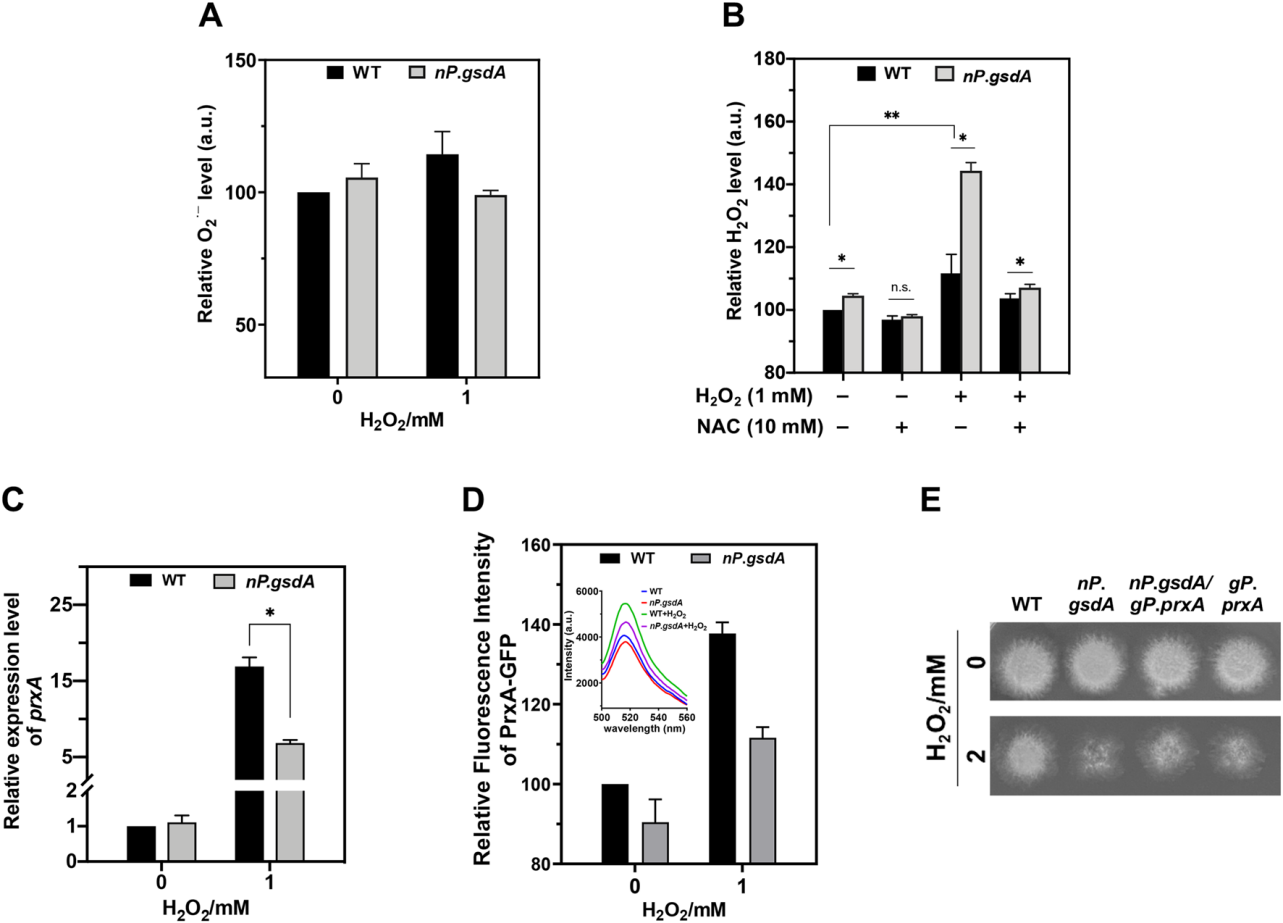
*prxA* suppression is responsible for the impairment of fungal resistance to H_2_O_2_ in G6PD-overexpression strains. All cultivations used NO_3_^−^ as the sole nitrogen source. **A**, **B** Quantification analysis of intracellular O_2_^·−^ and H_2_O_2_ in WT (WT_*pyrG*) and *nP*.*gsdA*. After precultivation, both strains were exposed to 0 or 1 mM H_2_O_2_ for 30 min followed by addition of ROS fluorescent probes. The ROS scavenger NAC (10 mM) was applied 1 h before the probe incubation. Fluorescence intensities of BES-So-AM and BES-H_2_O_2_-Ac were used to measure the level of intracellular superoxide and H_2_O_2_, respectively. All values were normalized by that in the unstressed WT (set to 100) (mean ± SD; *n* = 3, *P < 0.05, **P < 0.001, one-way ANOVA). **C** Relative expression levels of *prxA* in WT (WT_*pyrG*) and *nP*.*gsdA*. Strains were precultivated for 16 h, and then exposed to 1 mM H_2_O_2_ for 30 min. The level of *prxA* in WT without H_2_O_2_-treatment was set to 1 (mean ± SD; *n* = 3, *P < 0.05, one-way ANOVA). **D** Relative Prx-GFP levels in the WT (P_Gfp) and *nP*.*gsdA* (*nP*.*gsdA*/P_Gfp) strains. Inset, fluorescence spectra of Prx-GFPs from the corresponding cell lysates. After preculture, both strains were exposed to 0 and 1 mM H_2_O_2_ for 2 h. Cell lysates (1 mg/ml) were used for fluorescence analysis. **E** Effects of constitutive expression of *prxA* on fungal oxidative resistance. Conidia (1 × 10^5^) of the strains were spotted and cultivated for 2 days on NO_3_^−^-MM plates with or without 2 mM H_2_O_2_. Newly constructed strains are as follows: *gP*.*prxA* (replacing *prxA* promoter with *gpdA* promoter) and *nP*.*gsdA*/*gP*.*prxA* (replacing *gsdA* promoter with *niaD* promoter in *gP*.*prxA*)

To investigate whether excess NADPH increased the levels of oxidants, we used fluorescent probes to measure and compare superoxide and H_2_O_2_ accumulated in WT and *nP*.*gsdA* strains. Although excess NADPH theoretically can be utilized by NOXs to produce superoxide (Leopold et al. 2003; Gupte et al. 2007; Lee et al. 2011), overexpression of *A. nidulans gsdA* did not lead to an increase in intracellular superoxide under both stressed and unstressed conditions (Fig. 3A). However, a high level of NADPH appeared to directly contribute to the production of H_2_O_2_ because a slight but significant increase of H_2_O_2_ accumulation was detected in NO_3_^−^-induced *nP*.*gsdA* than that in WT under normal conditions (Fig. 3B). H_2_O_2_ exposure has further promoted intracellular H_2_O_2_ accumulation in both strains and enlarged the difference in H_2_O_2_ level between WT and *nP*.*gsdA* (Fig. 3B). Moreover, the elevation of H_2_O_2_ accumulation was prevented by the H_2_O_2_ scavenger N-acetyl-l-cysteine (NAC, 10 mM) (Fig. 3B), which also eliminated the H_2_O_2_-sensitivity difference between both strains (Additional file 1: Fig. S9). Therefore, it can be concluded that excess NADPH directly contributes to toxic level of H_2_O_2_ accumulation in fungal cells under oxidative stress conditions.

### Excess NADPH suppresses *prxA* transcription by downregulating NapA

Logically, intracellular H_2_O_2_ accumulation can be attributed to the inefficiencies of the key H_2_O_2_-decomposing enzymes. To explore whether excess NADPH impaired the antioxidant function of *A. nidulans* PrxA, we compared the transcriptional levels of *prxA* in WT and NO_3_^−^-induced *nP*.*gsdA*. As expected, external H_2_O_2_ greatly increased PrxA transcriptional levels in WT (Fig. 3C), which was consistent with previous findings (Thon et al. 2010; Xia et al. 2018). H_2_O_2_-induced *prxA* expression was also observed in NO_3_^−^-induced *nP*.*gsdA* strains; however, the induction strength was approximately 50% lower than that of WT (Fig. 3C). To investigate whether the transcriptional induction of *prxA* results of the corresponding changes of PrxA at protein level, we constructed GFP-tagged PrxA expression strains P_Gfp and *nP*.*gsdA*/P_Gfp, facilitating the quantification estimation of intracellular PrxA by fluorescence intensity measurements. The P_Gfp strain restored the oxidative resistance caused by *prxA* deletion (Additional file 1: Fig. S3), indicating the full function of Gfp-tagged PrxA. The same change tendency between gene transcription and protein expression was observed: the induction strength of PrxA in *nP*.*gsdA* was lower than in WT, which was indicated by the fluorescence intensity of PrxA-GFP in P_Gfp and *nP*.*gsdA*/P_Gfp under H_2_O_2_ treatment conditions (Fig. 3D). We hypothesized that the adverse induction of *prxA* expression caused by excess NADPH may account for the H_2_O_2_ accumulation and subsequent H_2_O_2_ defense defect in NO_3_^−^-induced *nP*.*gsdA*. To verify this, we constructed two *prxA*-constitutively expressing strains (*gP*.*prxA* and *nP*.*gsdA*/*gP*.*prxA*) using WT and *nP*.*gsdA* as parent strains, respectively (Additional file 1: Fig. S2), and analyzed their antioxidant abilities. In both strains, constitutive expression of *prxA* was realized by replacing the *prxA* promoter with *gpdA* promoter. As expected, constitutive expression of *prxA* abrogated the distinct of H_2_O_2_-resistance between *gP*.*prxA* and *nP*.*gsdA*/*gP*.*prxA*, which was in sharp contrast to WT and *nP.gsdA* (Fig. 3E). Collectively, these data further illustrate that NADPH may determine the antioxidant ability of fungi via regulating the gene transcription of PrxA, the frontline defender against H_2_O_2_.

**Fig. 4:**
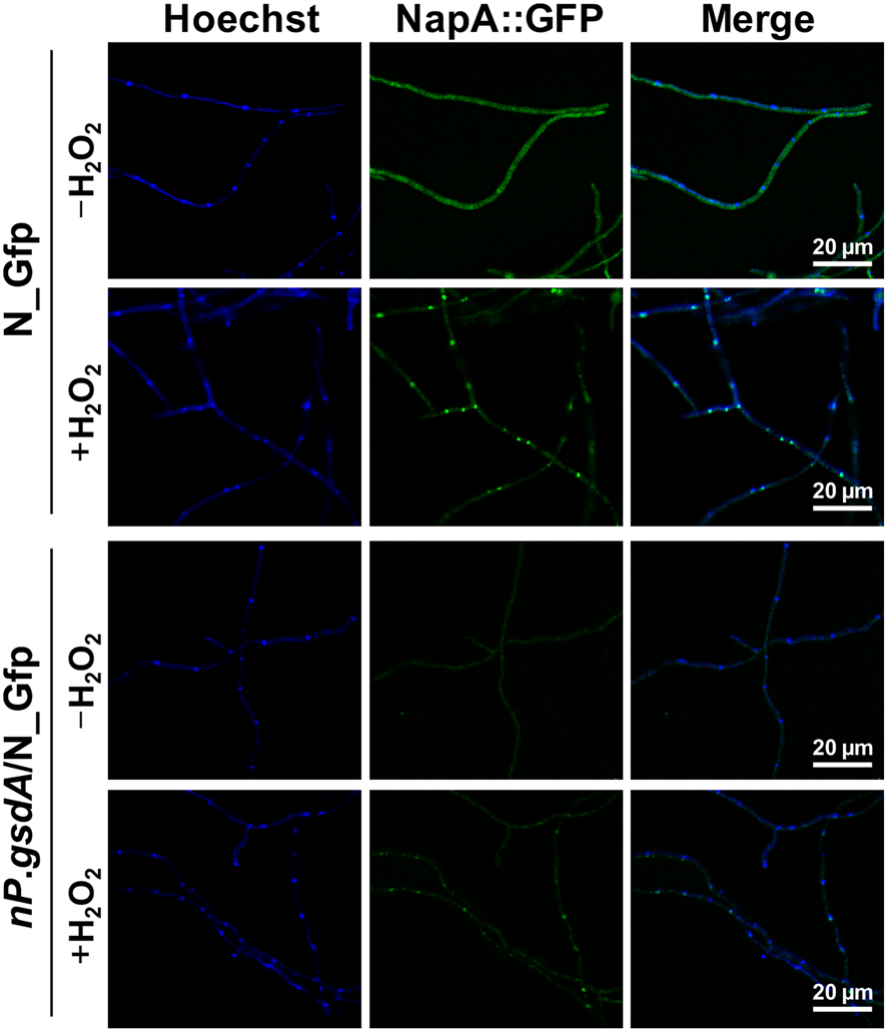
Overexpressing G6PD downregulated expression of NapA but did not interfere with its nuclear localization. Fresh conidia (1 × 10^5^) of N_Gfp (NapA::GFP) and *nP*.*gsdA*/N_Gfp (replacing *gsdA* promoter with *niaD* promoter in N_Gfp) was incubated in NO_3_^−^-MM for 10 h and was then exposed to 0 or 2 mM H_2_O_2_ for 20 min; nuclei were stained with Hoechst 33258 for 15 min. Scale bar, 20 μm. Images were captured using a laser confocal microscope

Repression of *prxA* transcription by accelerating intracellular NADPH production led us to infer that the function of NapA, the common transcriptional activator of fungal antioxidant genes, including *prxA*, is impaired under these conditions since NADPH should be the electron donor for NapA reduction and result in consequent deactivation of NapA (Thon et al. 2010). To validate this prediction, we first examined whether NapA can correctly localize in response to H_2_O_2_ exposure in the presence of excess intracellular NADPH. A GFP-tagged NapA was introduced to replace the original NapA in WT to construct N_Gfp (NapA-GFP) (Additional file 1: Fig. S3). The H_2_O_2_ resistance of N_Gfp was similar to that of WT (Additional file 1: Fig. S3), indicative of the functionality of this NapA::GFP fusion. The strain N_Gfp was further transformed with the *pyrG*-*niaD*.P-*gsdA* cassette to construct a new strain *nP*.*gsdA*/N_Gfp which can realize the NO_3_^−^-inducible overexpression of *gsdA* in the fluorescent strain. Then, we characterized NapA::GFP localization and found that H_2_O_2_ exposure quickly resulted in NapA::GFP nuclear accumulation in both strains, indicating that activation of NapA was not interfered by excess intracellular NADPH (Fig. 4). Surprisingly, we found that *nP*.*gsdA*/N_Gfp showed significantly reduced fluorescence intensity compared with that of N_Gfp regardless with or without the presence of H_2_O_2_ (Fig. 4), indicating that excess intracellular NADPH impaired NapA production, which occurred prior to H_2_O_2_ exposure. Thus, we demonstrated that excess intracellular NADPH modulates fungal antioxidant activity by downregulating the amount of NapA rather than by affecting the redox state of NapA.

### Excess NADPH obligatorily activates AnCF to repress *napA* expression

**Fig. 5:**
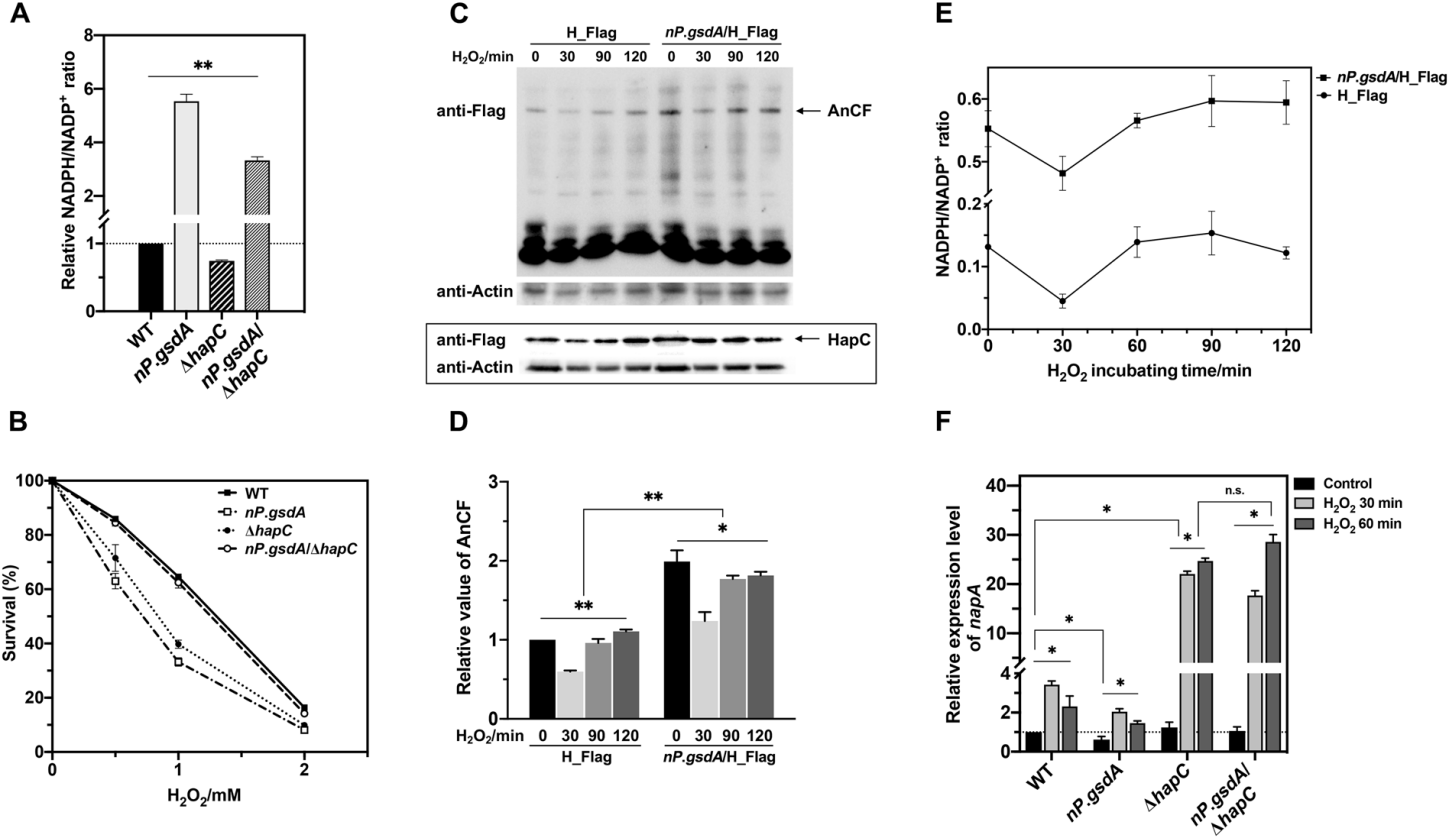
Intracellular NADPH levels determine the oxidative stress resistance via regulating AnCF complex assembly. **A** Overexpression of *gsdA* resulted NADPH accumulation in Δ*hapC*. WT (WT_*pyrG*), *nP*.*gsdA*, ∆*hapC*, and *nP*.*gsdA*/∆*hapC* were cultivated in NO_3_^−^-MM liquid media for 16 h, then the lysates were used for the relative NADPH/NADP^+^ ratio calculation. NADPH/NADP^+^ value of WT was set to 1 (mean ± SD; *n* = 3, **P < 0.01, one-way ANOVA). **B** AnCF is involved in impairment of H_2_O_2_ resistance caused by excess NADPH. Fresh conidia (1 × 10^8^) of four strains were spread on NO_3_^−^-MM plates containing the indicated concentrations of H_2_O_2_. Colonies were counted after a 48-h incubation, and survival rates were expressed as percentages of the CFU for strains incubated without H_2_O_2_ (mean ± SD; *n* = 3). **C** Immunoblot quantification of AnCF complex levels (top) and HapC levels (bottom box) in WT and *nP*.*gsdA* during H_2_O_2_-treatment; H_Flag (WT expressing Flag-tagged HapC) and *nP*.*gsdA*/H_Flag (*nP*.*gsdA* expressing Flag-tagged HapC). Cell-free lysates (100 µg) from each sample were loaded to native- (top) and SDS-PAGE (bottom box). AnCF and HapC were detected using anti-Flag antibody; actin was used as a control and was detected using an anti-Actin antibody. **D** Quantitated graph for intracellular AnCF level normalized to actin (mean ± SD; *n* = 3, *P < 0.05, one-way ANOVA). **E** Time course analysis of NADPH/NADP^+^ level in H_Flag and *nP*.*gsdA*/H_Flag during 1 mM H_2_O_2_ treatment. Strains were precultivated in NO_3_^−^-MM liquid media for 16 h and then exposed to 1 mM H_2_O_2_ for 30, 60, and 90 min (mean ± SD; *n* = 3, *t*-test). **F** Relative *napA* mRNA levels in these strains with or without treatment of H_2_O_2_. Strains were cultivated in NO_3_^−^-MM liquid media for 16 h, and then treated by H_2_O_2_ for 30 min and 60 min (mean ± SD; *n* = 3, *P < 0.05, **P < 0.01, n.s*.*, not significant, two-way ANOVA)

To elucidate the mechanism whereby NapA expression was downregulated by excess NADPH, we focused on the dynamics of the levels of AnCF, which is a key transcriptional repressor of NapA (Thon et al. 2010; Hortschansky et al. 2017). The HapB, HapC, and HapE subunits of AnCF are all necessary for DNA binding (Thon et al. 2010; Hortschansky et al. 2017). AnCF senses the redox status of the cell via oxidative modification of thiol groups within HapC; oxidized HapC is then unable to participate in AnCF assembly, but can be reduced by the thioredoxin system (TrxA and TrxR) for recycling in the AnCF assembly. Thus, we questioned if excess NADPH can over-reduce HapC, leading to the ROS-resistant defect of *nP*.*gsdA*. If this is the case, deletion of *hapC* should relieve fungal H_2_O_2_ sensitivity caused by excess NADPH. Thus, we have constructed a *hapC* deletion strain (∆*hapC*) (Additional file 1: Fig. S1) and overexpressed G6PD in this mutant (*nP*.*gsdA*/∆*hapC*) to understand the relationship among HapC, excess NADPH, and cell H_2_O_2_ resistance. Deletion of *hapC* resulted in a great growth defect under normal conditions (Additional file 1: Fig. S4), which is consistent with previous reports (Papagiannopoulos et al. 1996). Overexpression of *gsdA* using *niaD*.P under NO_3_^−^ conditions also realized excess NADPH accumulation in *nP*.*gsdA*/∆*hapC* (Fig. 5A). Next, we compared conidia survival rates of WT, *nP*.*gsdA*, ∆*hapC*, and *nP*.*gsdA*/∆*hapC* strains in response to oxidative stress induced by H_2_O_2_ under NO_3_^−^ induction (Fig. 5B). The ∆*hapC* strain showed significantly decreased survival rate in all H_2_O_2_ stress conditions compared with that of the WT strain, indicating that AnCF is indispensable to *A. nidulans* oxidative stress resistance. Notably, overexpressing *gsdA* in ∆*hapC* did not impair fungal oxidative stress resistance, and, in contrast, substantially rescued the survival rate of *nP*.*gsdA*/∆*hapC* strain (Fig. 5B), clearly indicating that, in the absence of AnCF, extra NADPH supply is advantageous for fungal ROS defense. That is to say, impairment of the antioxidant ability caused by excess NADPH in WT is mediated by the AnCF complex.

Next, we obtained insight into the relevance between the level of intracellular NADPH and AnCF complex assembly. The Flag-tagged HapC was introduced into both WT and *nP*.*gsdA* strains to replace native HapC and construct H_Flag and *nP*.*gsdA*/H_Flag strains, respectively (Additional file 1: Fig. S4), enabling the measurement of the cellular level of AnCF in both strains by western blotting. We first confirmed that HapC-Flag protein in both H_Flag and *nP*.*gsdA*/H_Flag strains complemented the growth delay caused by *hapC* deletions (Additional file 1: Fig. S4), indicating that the fusion protein was functional. No bands were detected in cells expressing untagged HapC in WT (data not shown). Considering that the assembly and dissociation of AnCF may affect the dynamics of the intracellular AnCF content, we measured the content of AnCF with time. The HapC bands on reducing SDS-PAGE showed that the total amount of HapC in WT cells was relatively stable across the 120-min observation period under H_2_O_2_-treatment conditions (Fig. 5C, bottom box, and Additional file 1: Fig. S10), while levels of AnCF complex in non-reducing native PAGE fluctuated with time (Fig. 5C, top and Fig. 5D). During the earlier 30 min of H_2_O_2_ exposure, the intracellular AnCF level declined to 2/3 the level of the pretreatment sample in H_Flag strain. Extending the H_2_O_2_ exposure time to 90 and 120 min has gradually recovered and stabilized the AnCF formation to the original level. Interestingly, we found that changes in the level of intracellular NADPH in H_Flag strain kept pace with the fluctuation of AnCF: a sudden drop in the first 30 min, which then returned to the original level within the next 60 min (Fig. 5E). Thus, we deduced that the NADPH intracellular contents may determine the level of the AnCF complex. This supposition was further supported by investigating the AnCF and intracellular NADPH profiles in NO_3_^−^-induced *nP*.*gsdA*/H_Flag, which was proved to be very similar to those present in H_Flag (Fig. 5C–E); moreover, the NADPH level in *nP*.*gsdA*/H_Flag was found to be well above that in H_Flag at any time (Fig. 5E). In response to the elevated NADPH, AnCF content also keeps higher in *nP*.*gsdA*/H_Flag than that in H_Flag (Fig. 5D). These data, taken together, showed that the initial “down then up” fluctuations of NADPH levels and AnCF contents are the first response of the fungal cells to the H_2_O_2_ stimulus.

We further deduced that the “down then up” fluctuation of AnCF content would result in a reverse fluctuation of NapA levels and corresponding up- and downregulated expression of *A. nidulans prxA*. This was verified by the following transcriptional changes of *napA* in strains upon H_2_O_2_ treatment (Fig. 5F). Exposure to H_2_O_2_ for the first 30 min induced *napA* expression in WT and *nP.gsdA*, as opposed to the downregulation of intracellular AnCF level in both strains (Fig. 5F). Notably, deletion of *hapC* drastically elevated *napA* induction amplitude compared with that of WT during the first 30 min of H_2_O_2_ exposure, confirming the transcriptional repression effect of AnCF on *napA*. Extending H_2_O_2_ exposure from 30 to 60 min decreased *napA* transcription in WT and *nP*.*gsdA* (Fig. 5F), which contrasted with the changes in the levels of AnCF (Fig. 5C, top). Conversely, in ∆*hapC* and *nP*.*gsdA*/∆*hapC*, extending H_2_O_2_ exposure from 30 to 60 min did not lower *napA* transcription levels (Fig. 5F), further confirming the involvement of AnCF in the negative regulation of *napA*. Since NapA is the transcription activator of *prxA*, the “down then up” content fluctuation of NADPH should be ultimately used to trigger and subsequently break the induction of *prxA* to provide the on-demand cellular level of PrxA for oxidative stress defense in *A. nidulans*.

### Reversible inhibition of G6PD may account for the NADPH fluctuation

Under oxidative stress conditions, a sudden “down” of intracellular NADPH level at the initial stage should be the result of NADPH consuming by PrxA for fungal antioxidant machinery. The following “up” of NADPH content suggests a quick activity acceleration of the NADPH-producing enzyme. *A. nidulans* G6PD may act as the key enzyme, because G6PDs from other sources have been reported to be reversibly inhibited by NADPH, which can be broken by rapid withdrawal of NADPH (Ramos-Martinez 2017). To verify that, we prepared recombinant G6PD of *A. nidulans* to test the NADPH-dependent inhibition and disinhibition of fungal G6PD in vitro. As shown in Additional file 1: Fig. S5, premixing G6PD with NADPH effectively inhibited fungal G6PD activity, which is indicative of self-braking of G6PD by its product NADPH. Next, we have investigated the disinhibition of G6PD by employing *A. oryzae* flavohemoglobin (a NADPH-dependent nitric oxide dioxygenase) (Zhou et al. 2011) as an NADPH scavenger. A rapid G6PD activation was achieved by the addition of flavohemoglobin and nitric oxide release reagent to the reaction buffer (Additional file 1: Fig. S5), indicating that regulation of *A. nidulans* G6PD activity is dependent on disinhibition. Taken together, these results supported our view that the most possible mechanism of NADPH fluctuation may be the result of the rapid NADPH consumption by PrxA upon oxidative exposure coupling the subsequent regeneration of NADPH via the disinhibition of G6PD. Moreover, the fungus may take advantage of the fluctuation of intracellular NADPH to regulate AnCF assembly in response to oxidative stress.

## Discussion

**Fig. 6:**
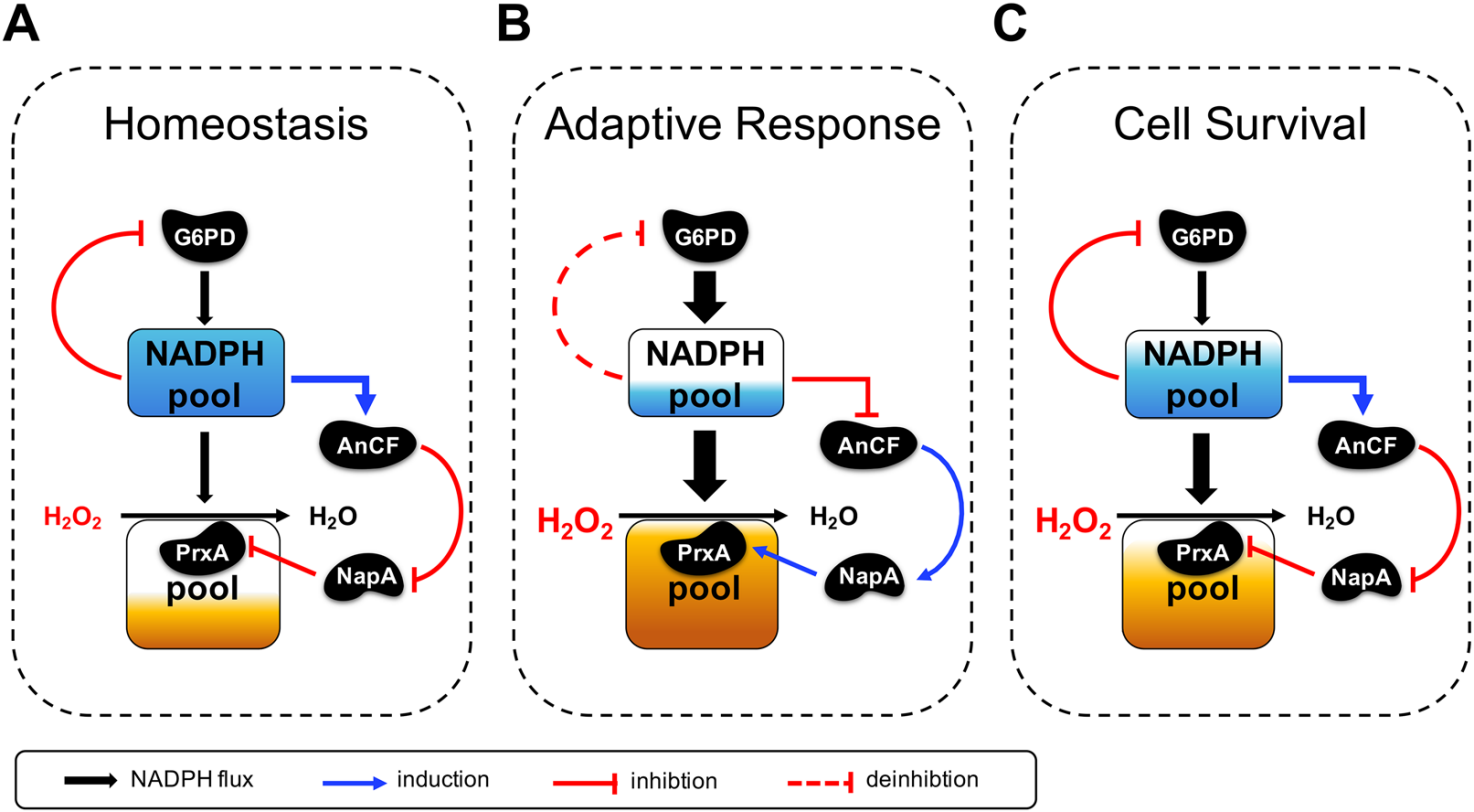
Schematic illustration of NADPH-mediated autoregulation mechanism for adaptation to oxidative stress in *A. nidulans*. **A**–**C** Under normal conditions (**A**, Homeostasis stage) the NADPH pool provides an adequate supply of NADPH to reduce AnCF to its activated state, which will suppress NapA and subsequently lower the levels of PrxA. Due to the absence of high consumption of NADPH, the intracellular NADPH pool is maintained at a high level causing G6PD activity inhibition. Thus, the inflow of NADPH from G6PD to the NADPH pool is quite limited. Once fungi encounter H_2_O_2_ (**B**, Adaptive response stage), NADPH is suddenly needed to decompose H_2_O_2_ using PrxA, leading to an immediate drop within 30 min. Inevitably, the inflow of NADPH from the NADPH pool to reduce AnCF is declined. The accumulation of oxidized AnCF activates *napA* to supply more PrxA until the PrxA pool is saturated, accelerating NADPH consumption while resisting H_2_O_2_. Next, the depletion of NADPH reactivates G6PD, and NADPH eventually reaches to the initial level in the next 30 min (**C**, Cell survival stage). The increased NADPH level triggers the reactivation of AnCF, which consequently shuts down NapA induction, represses NapA activity via NADPH-dependent reduction to block PrxA induction, and ultimately keeps the PrxA pool at a moderate level (**C**, Cell survival stage)

In *A. nidulans*, NADPH acts as a major reducing equivalent to reduce intracellular ROS, thus in this study, deficiency of NADPH, leads to low fungal viability under oxidative stress as expected. However, an increasing NADPH levels also impairs cellular tolerability to H_2_O_2_. Basing on our experiments, we reasoned for the unexpected phenotype as follows: excess NADPH promotes the assembly of AnCF, which in turn suppressed NapA expression, leading to low levels of PrxA and the eventual impairment of antioxidant ability. Moreover, the “down then up” fluctuation of the intracellular NADPH level, together with reversible inhibition activity of the fungal G6PD, lead us to deduce that the rhythm of NADPH may be an efficient survival strategy adapted by *A. nidulans* to defend against H_2_O_2_, as indicated in the proposed model (Fig. 6): the initial sudden decrease of NADPH resulting from PrxA-dependent H_2_O_2_-decomposition triggers the expression of *prxA* via the cascade regulation composed of AnCF and NapA. The subsequent recovery of NADPH allows this to shut down the further induction of *prxA*, which may be essential to maintain reasonable utilization of NADPH for other pathways under ROS stress conditions.

In animals, NADPH also serves as an important participant in maintaining cellular redox homeostasis. However, the effects of intracellular NADPH accumulation on cell fates are not always the same case. In some cells, increased NADPH levels were found to be beneficial in protecting against oxidative stress. For example, overexpression of G6PD decreases ROS accumulation in response to exogenous and endogenous oxidant in vascular endothelial cells (Leopold et al. 2003) and in aldosterone-treated bovine aortic endothelial cells (Leopold et al. 2007). Additionally, overexpression of G6PD was reported to extend the life span of transgenic *D. melanogaster* (Legan et al. 2008). Furthermore, G6PD induction was reported to be instrumental in regenerating the intracellular GSH pool in human HeLa cells, which may facilitate the cellular protection against oxidant injuries (Salvemini et al. 1999) as GSH is a critical antioxidant and scavenges ROS directly or as cofactor of the glutathione and thioredoxin systems in animal cells (Stincone et al. 2015). The most notable difference in ROS-defense system between *A. nidulans* and animals is that fungal GSH is not essential in H_2_O_2_ defense (Sato et al. 2011), which may be one reason for the different phenotypes between *A. nidulans* and animals triggered by excess intracellular NADPH under oxidative stress conditions. On the other hand, the accumulation of intracellular NADPH by overexpression of G6PD can result in reductive stress and ultimately ROS production (Xiao et al. 2018, 2020). For example, overexpression of G6PD upregulated mRNA expression of NOX gp91phox and p22phox subunits, potentiating ROS production and oxidative damage in mouse pancreatic β cells and thymic lymphoma cells, which provided evidences for NOX-mediated oxidative stress by excess intracellular NADPH (Tome et al. 2006; Lee et al. 2011). Upregulated G6PD expression also increases oxidative stress in human tissue, such as failing human heart (Gupte et al. 2007). The adverse effects of NADPH on ROS damage protection have been interpreted as excess NAD(P)H being used by NOXs to produce ROS (Bedard et al. 2007; Sarsour et al. 2009; Handy et al. 2012). However, excess NADPH did not facilitate the intracellular O_2_^·−^ accumulation in *A. nidulans* (Fig. 3A). Therefore, animals do not apparently share the same mechanism with fungi of a NOX-mediated increase of ROS triggered by excess NADPH. The homologs of AnCF and NapA are highly conserved in many filamentous fungi and yeasts, including most of the *Aspergillus* species (Brakhage et al. 1999; Zheng et al. 2015; Hortschansky et al. 2017; Mendoza-Martinez et al. 2017), *S. cerevisiae* (McNabb et al. 1995; Rodrigues-Pousada et al. 2019), *S. pombe* (McNabb et al. 1997; Boronat et al. 2014), *Kluyveromyces lactis* (Mulder et al. 1994; Imrichova et al. 2005) and *Cryptococcus neoformans* (Loussert et al. 2010; Pais et al. 2016). While systematic studies remain lacking, the striking mechanistic similarities in ROS defense between *A. nidulans* and these fungi indicate that utilizing NADPH fluctuation to assure timely activation and avoid overactivation of the key antioxidants as an oxidative adaptation strategy may be conserved among these fungi.

Is it necessary for *A. nidulans* to regulate the activity of PrxA in a feedback manner? If there was not a NADPH-mediated genetic regulation cascade between PrxA and AnCF as well NapA, the continued presence of H_2_O_2_ will result in substantial PrxA production. The accumulated PrxA will deplete the intracellular NADPH for its ROS decomposition reaction, depriving NADPH from other important NADPH-utilizing cell metabolisms such as the repair of oxidized proteins (Lu et al. 2014), fatty acid synthesis, reductive assimilation of inorganic sulfur (Thomas et al. 1991), and restoration of cellular pools of reduced glutathione and thioredoxin (Miller et al. 2018), which may lead to more cellular damage. Thus, as a double-edged sword in response to oxidative stress, *prxA* should be expressed on-demand. A similar case was observed in *Schizosaccharomyces pombe* (Day et al. 2012; Brown et al. 2013). At high levels of H_2_O_2_, which are acute stressful to the yeast, inactivating Tpx1 (a PrxA homolog) by hyperoxidation to a Trx1-resistant sulfinic (SOOH) derivative was essential to target reduced Trx1 toward other substrates, allowing the repair of oxidized proteins vital for cell survival under these conditions. In fact, inactivating Tpx1 to save reduced Trx1 is tantamount to saving NADPH since reduced Trx1 is derived from NADPH-dependent reduction in yeasts. Notably, the *A. nidulans* PrxA is a hyperoxidation-resistant peroxidase that can resist extremely high concentrations of H_2_O_2_ (Xia et al. 2018). Thus, PrxA cannot shift NADPH toward other substrates via self-inactivation by a high concentration of H_2_O_2_ as what occurs with yeast Tpx1. Alternatively, the balance of NADPH supply to H_2_O_2_-defense system and other metabolism in *A. nidulans* may be realized via the NADPH-mediated feedback mechanism.

NADPH-dependent metabolic reactions have been identified to be indispensable tools in biomanufacturing. A vast number of important targets including most natural products, amino acids, fatty acids, nucleotides, sterols and steroids are synthesized via NADPH-dependent pathways (Yu et al. 2018; Zhang et al. 2018; Gu et al. 2021). Furthermore, NADPH serves as the preferred electron donor for some of the largest and most versatile classes of enzymes such as cytochrome P450 enzymes and enoate reductases, which have broad applications in metabolic engineering. In these systems, the presence of NADPH-consuming reactions lowers the intracellular NADPH level and decreases the desired reaction rate, which may cause defective effects to cells; therefore, elevating G6PD enzymatic activity to enhance NADPH supply was a widely adopted strategy for metabolic engineering. Filamentous fungi are arguably the most industrially important group of microorganisms. Production processes involving these simple eukaryotes are often highly aerobic in nature, which implies that cultures are routinely subject to oxidative stress (Gibbs et al. 2000; Li et al. 2011). Thus, supply of more intracellular NADPH by overexpressing G6PD to improve fermentation products may be challenging because of the possible oxidative damage caused by the excess intracellular NADPH. However, the coincidental overexpression of G6PD and PrxA can partially alleviate oxidative damage (Fig. 3E), leading us to hypothesize that further the elevating cellular PrxA level by a genetic method may cover the defective effects produced by G6PD overexpression on ROS resistance and ultimately contribute to NADPH-demanding production synthesis.

## Conclusion

Increasing intracellular NADPH promotes the assembly of the CCAAT-binding factor AnCF, which in turn suppressed NapA, a transcriptional activator of the key ROS scavenger PrxA, leading to low levels of PrxA and the eventual impairment of antioxidant ability in *Aspergillus nidulans*.

### Supplementary Information

**Additional file 1: Table S1.** *A. nidulans* strains used in this study. **Table S2.** Primers used in this study. **Fig. S1.** CRISPR/Cas9-mediated disruptions of *hapC* gene in *A. nidulans*. **A** Schematic diagram of gene disruption by in vitro assembled Cas9/sgRNA and donor DNAs. **B** Gene replacement of the target gene loci via the homology-directed repair pathway. Left, Schematic diagram of locus changes of target genes before and after gene replacement. Right, Confirmation of gene disruptions by PCR using the indicated primer pairs. *hapC* disruptant was verified with primer pairs *AN4034*-uF/*pyrG*-check-5′-R (lane 1), *AN4034*-dR/*pyrG*-check-3′-R (lane 2), respectively; primer pair *hapC*-check-F/*hapC*-check-R was used to amplify AN4034 ORF in the parent (lane 3) and disruptant (lane 4) strains. M, marker. Primers are listed in Table S2. **Fig. S2.** Constructions of promoter substitution strains of *A. nidulans*. **A**–**D** Conditional promoter replacement strategy for homologous recombinant strains *nP*.*gsdA* (A), *gP*.*gsdA* (B), *gP*.*gndA* (C), *gP*.*prxA* (D) and validation of the corresponding recombinations by PCR with the indicated primers (right). The isolated *nP*.*gsdA* transformant was verified with primer pairs *AN2981*-uF/*pyrG*-check-5′-R (lane 1), *AN2981*-dR/*pyrG*-check-3′-R (lane 2), respectively; Primer pair *AN2981*-uF/*AN2981*-dR was used to amplify corresponding regions in the transformant (lane 3) and parent (lane 4) strains. M, marker. The similar methods were performed to validate the resultant transformants of *gP*.*gsdA* and *gP*.*gndA*. For verifying the indicated recombination in *gP*.*prxA*, primer pair M13-R/*pyroA*-check-3′-F was used to amplify corresponding regions in the transformant (lane 3) and parent (lane 4) strains. Primers are listed in Table S2. **Fig. S3.** Construction and phenotype analysis of GFP-tagged NapA strain and GFP-tagged PrxA strain. **A** and **C** Schematic diagram of locus changes of *napA* gene (A) and *prxA* gene (C) before and after the gene replacements (left) and the corresponding confirmation using PCR (right). Primer pair *napA*-5′-F/*napA*-3′ UTR-R was used to amplify the corresponding region in the isolated transformant (lane 1) and the parent (lane 2) strains. M, marker. Primers are listed in Table S2. **B** GFP-tagged NapA strain (N_Gfp) shows similar H_2_O_2_ resistance to the control strain (WT_*argB*). Conidia (1 × 10^5^) from both strains were spotted on MM plates with or without 2 mM H_2_O_2_ and incubated at 37 °C for 2 days. **D** GFP-tagged GFP strain (P_Gfp) recovered the H_2_O_2_ resistance and similar to the control strain (WT_*argB*). Conidia (1 × 10^5^) from both strains were spotted on MM plates with or without 1 mM H_2_O_2_ and incubated at 37 °C for 2 days. **Fig. S4.** Construction of Flag-tagged HapC strain and phenotype analysis of variants of *hapC* mutant under unstressed conditions. **A** The recombinant plasmid pUC19-*pyroA*-*hapC*-Flag was used to transform to *∆hapC* to construct H_Flag strain. The indicated recombinant transformant was isolated and further confirmed by PCR with the primer pair M13-R/*pyroA*-check-3′-F (right, lane 1). Lane 2 showed the corresponding result of the control strain. Primer are listed in Table S2. **B** Conidia (1 × 10^5^) from the control (WT_*argB*), *∆hapC*, *nP*.*gsdA/∆hapC* and HapC::Flag fusion protein-expressing strains H_Flag, and *nP*.*gsdA*/H_Flag were spotted on MM plate without H_2_O_2_ and incubated at 37 °C for 2 days. **Fig. S5.** Reversible inhibition of NADPH to G6PD activity. **A** SDS-PAGE (12%) analysis of purified recombinant AnG6PD expressed by *E. coli*. M, maker. Lane 1, purified AnG6PD. **B** Activity of recombinant G6PD samples was estimated by generation of NADPH using a UV–Vis spectrophotometer at 340 nm. G6PD activity was measured in a 1 ml reaction mixture containing 5 mM glucose-6-phosphate, 0.3 mM NADP^+^, and 5 µM G6PD; 30 µM NADPH was added to the reaction mixture to evaluate the inhibition effects on G6PD activity. Further addition of 20 µM flavohemoglobin (Fhb1), and 50 µM NO donor MAHMA NONOate was used to consume NADPH for inhibition relief of G6PD activity. **Fig. S6.** Downregulation of *A. nidulans gsdA* is detrimental to fungal growth and oxidative stress resistance. **A** Survival rates of WT (WT_*pyrG*) and *nP*.*gsdA* on MM plates using ammonium tartrate as nitrogen source under oxidative stress conditions. Fresh conidia (1 × 10^8^) of both strains were spread on MM plates containing the indicated concentrations of H_2_O_2_. Colonies were counted after a 48-h incubation, and survival rate are expressed as percentages of the CFU for strains incubated without H_2_O_2_. **B**–**C** G6PD activities and the relative NADPH/NADP^+^ ratio in WT and NH_4_^+^-repressed *nP*.*gsdA* strains before and after treatment of H_2_O_2_. Both strains were cultivated in MM liquid media using ammonium tartrate as the nitrogen source for 16 h, and then treated with the indicated concentrations of H_2_O_2_ for 30 min. (mean ± SD; n = 3, *P < 0.05, **P < 0.01, ***P < 0.001; n.s*.*, not significant, one-way ANOVA.) **Fig. S7.** Replacement of *gsdA* native promoter with *gpdA* promoter also perturbed NADPH rhythm and impaired fungal resistance to H_2_O_2_. **A**–**B** Relative expression levels of *gsdA* (A) and NADPH/NADP^+^ ratios (B) in WT (WT_*pyrG*), *nP*.*gsdA* and *gP*.*gsdA* (replacing *gsdA* promoter with *gpdA* promoter in WT) strains. All strains were precultivated in liquid NO_3_^–^-MM for 16 h and then exposed to 1 mM H_2_O_2_ for 30 min. The WT level of *gsdA* was set to 1, and the levels of *gsdA* in other strains were normalized to this. **C** Comparison of the H_2_O_2_ resistance of WT (WT_*pyrG*), *nP*.*gsdA* and *gP*.*gsdA*strains. (mean ± SD; n = 3, *P < 0.05, **P < 0.001, *t*-test.) **Fig. S8.** Enhancing NADPH by overexpression of *gndA* impairs fungal resistance to H_2_O_2_. **A**–**B** Relative expression levels of *gndA* (A) and NADPH/NADP^+^ ratios (B) in WT (WT_*pyrG*) and *gP*.*gndA* (replacing *gndA* promoter with *gpdA* promoter in WT) strains. All strains were precultivated in liquid NO_3_^–^-MM for 16 h and then exposed to 1 mM H_2_O_2_ for 30 min. The WT level of *gndA* was set to 1. **(C)** Comparison of the H_2_O_2_ resistance of WT (WT_*pyrG*) and *gP*.*gsdA* strains. (mean ± SD; n = 3, *P < 0.05, **P < 0.001, *t*-test.) **Fig. S9.** Intracellular H_2_O_2_ accumulation accounts for the growth retardation of *nP.gsdA* strain under oxidative stress conditions. Growth comparison of WT (WT_*pyrG*) and *nP*.*gsdA* strains under oxidative stresses or unstressed conditions. Conidia (1 × 10^5^) from both strains were spotted and cultivated for 2 days on NO_3_^–^-MM plates supplied with or without 10 mM NAC and 1 mM H_2_O_2_ as indicated by “+” and “−”, respectively. **Fig. S10.** Quantitative analysis of relative levels of intracellular HapC. Total contents of intracellular HapC levels of WT and *nP*.*gsdA* strains at different periods are shown by the intensity of bands on denaturing and reducing PAGE. HapC levels were normalized to actin contents calculated by the same method for the further quantitative comparison. Each value represents the mean ± SD of triplicate determinations (mean ± SD; *P < 0.05, **P < 0.01, one-way ANOVA).

## Data Availability

The data and the materials are all available in this article as well as the Additional file 1.

## Abbreviations

ROS: Reactive oxygen species
G6PD: Glucose-6-phosphate dehydrogenase
NADPH: Nicotinamide adenine dinucleotide phosphate
Prx: Peroxiredoxin
Trx: Thioredoxin
TrxR: Thioredoxin reductase
6PGD: 6-Phosphogluconate dehydrogenase
GR: Glutathione reductase
NOX: NADPH oxidase
