# Supplementary material for: Increasing NADPH impairs fungal H2O2 resistance by perturbing transcriptional regulation of peroxiredoxin
Source: Bioresour Bioprocess. 2022 Jan 3;9(1):1. doi: 10.1186/s40643-021-00489-w (PMC10992141; doi:10.1186/s40643-021-00489-w)
Supplement: Supplementary file 1 — Additional file 1: Table S1. A. nidulans strains used in this study. Table S2. Primers used in this study. Fig. S1. CRISPR/Cas9-mediated disruptions of hapC gene in A. nidulans. A Schematic diagram of gene disruption by in vitro assembled Cas9/sgRNA and donor DNAs. B Gene replacement of the target gene loci via the homology-directed repair pathway. Left, Schematic diagram of locus changes of target genes before and after gene replacement. Right, Confirmation of gene disruptions by PCR using the indicated primer pairs. hapC disruptant was verified with primer pairs AN4034-uF/pyrG-check-5′-R (lane 1), AN4034-dR/pyrG-check-3′-R (lane 2), respectively; primer pair hapC-check-F/hapC-check-R was used to amplify AN4034 ORF in the parent (lane 3) and disruptant (lane 4) strains. M, marker. Primers are listed in Table S2. Fig. S2. Constructions of promoter substitution strains of A. nidulans. A–D Conditional promoter replacement strategy for homologous recombinant strains nP.gsdA (A), gP.gsdA (B), gP.gndA (C), gP.prxA (D) and validation of the corresponding recombinations by PCR with the indicated primers (right). The isolated nP.gsdA transformant was verified with primer pairs AN2981-uF/pyrG-check-5′-R (lane 1), AN2981-dR/pyrG-check-3′-R (lane 2), respectively; Primer pair AN2981-uF/AN2981-dR was used to amplify corresponding regions in the transformant (lane 3) and parent (lane 4) strains. M, marker. The similar methods were performed to validate the resultant transformants of gP.gsdA and gP.gndA. For verifying the indicated recombination in gP.prxA, primer pair M13-R/pyroA-check-3′-F was used to amplify corresponding regions in the transformant (lane 3) and parent (lane 4) strains. Primers are listed in Table S2. Fig. S3. Construction and phenotype analysis of GFP-tagged NapA strain and GFP-tagged PrxA strain. A and C Schematic diagram of locus changes of napA gene (A) and prxA gene (C) before and after the gene replacements (left) and the corresponding confirmation using [file 40643_2021_489_MOESM1_ESM.docx]

**Additional file 1**

**Increasing NADPH impairs fungal H_2_O_2_ resistance by perturbing transcriptional regulation of peroxiredoxin**

Jingyi Li^1^, Yanwei Sun^1^, Feiyun Liu^1^, Yao Zhou^1^, Yunfeng Yan^1^, Zhemin Zhou^2^, Ping Wang ^3,^*, and Shengmin Zhou ^1,^*

There are 2 supplementary tables and 10 supplementary figures associated with this manuscript.

**Table S1. *A. nidulans* strains used in this study.**

| Strain | Genotype | Source |
| --- | --- | --- |
| ABPUN | *biA1*; *pyrG89*; *wA3*; *argB2*; *pyroA4* | Our lab |
| A6 | *nicA2* (*veA*+) | FGSC |
| WT_*pyrG* | *biA1*; *wA3*; *argB2*; *pyroA4*; *AopyrG*+ | This work |
| WT_*argB* | *biA1*; *pyrG89*; *wA3*; *pyroA4*; *AnargB*+ | This work |
| *∆prxA* | *∆prxA*; *biA1*; *pyrG89*; *wA3*; *pyroA4*; *AnargB*+ | Our lab |
| *nP*.*gsdA* | *biA1*; *wA3*; *argB2*; *pyroA4*; *niaD.*P*-gsdA*; *AopyrG*+ | This work |
| P_Gfp | *prxA*-*gfp*:: *∆prxA*; *biA1*; *pyrG89*; *wA3*; *AnpyroA*+; *AnargB*+ | This work |
| *nP*.*gsdA*/P_Gfp | *prxA*-*gfp*:: *∆prxA*; *biA1*; *wA3*; *niaD.*P*-gsdA*; *AopyrG*+; *AnpyroA*+; *AnargB*+ | This work |
| N_Gfp | *napA*::*gfp*; *biA1*; *pyrG89*; *wA3*; *AnpyroA*+; *AnargB*+ | This work |
| *nP*.*gsdA*/N_Gfp | *napA*::*gfp*; *biA1*; *wA3*; *AnpyroA*+; *AnargB*+; *niaD.*P*-gsdA*; *AopyrG*+ | This work |
| *gP*.*gsdA* | *biA1*; *wA3*; *argB2*; *pyroA4*; *gpdA*.P*-gsdA*; *AopyrG*+ | This work |
| *gP*.*gndA* | *biA1*; *wA3*; *argB2*; *pyroA4*; *gpdA*.P*-gndA*; *AopyrG*+ | This work |
| *∆hapC* | *∆hapC*; *biA1*; *pyrG89*; *wA3*; *pyroA4*; *AnargB*+ | This work |
| *nP*.*gsdA*/*∆hapC* | *∆hapC*; *biA1*; *wA3*; *pyroA4*; *AnargB*+; *niaD.*P*-gsdA*; *AopyrG*+ | This work |
| H_Flag | *hapC*-Flag::*∆hapC*; *biA1*; *pyrG89*; *wA3*; *AnargB*+ | This work |
| *nP*.*gsdA*/H_Flag | *hapC*-Flag::*∆hapC*; *biA1*; *wA3*; *pyroA4*; *AnargB*+; *niaD.*P*-gsdA*; *AopyrG*+ | This work |
| *gP*.*prxA* | *biA1*; *wA3*; *argB2*; *pyroA4*; *gpdA.*P*-prxA*; *AopyrG*+ | This work |
| *nP*.*gsdA*/*gP*.*prxA* | *biA1*; *wA3*; *argB2*; *pyroA4*; *gpdA.*P*-prxA*; *niaD*.P*-gsdA*; *AopyrG*+ | This work |

**Table S2**. Primers used in this study.

| Primers | Gene | Nucleotide Sequence | Used for |
| --- | --- | --- | --- |
| **Gene Disruption** |  |  |  |
| *pyrG*-F1 | *Ao.pyrG* | ATGCGAAGGTAAGTGCTTC | Selective maker gene |
| *pyrG*-R |  | TGGCTAGGCTCTGACTCGATG |  |
| *pyroA*-F | *An.pyroA* | GGCATGGATGAACTATACAAATAACTGCAGAAGTGCGCGAAAG |  |
| *pyroA*-R |  | GCTCTGGACTGTGCCATG |  |
| *argB*-F | *An.argB* | AAGCTTTATTTCGCGGTTTTTTG |  |
| *argB*-R |  | GTCGACCTACAGCCATTGCG |  |
| *AN**4034*-uF | *hapC* | CTTGACTAGTTCGTCAAGC | Δ*hapC* |
| *AN4034*-uR |  | GACTACCCCAAAAAACCGCGAAATAGTTTTAGCTGTTGGCGAAGGTG |  |
| *AN4034*-dF |  | GGTTTCGCAATGGCTGTAGGTCGACTCTTTCTGTTACGGAAATG |  |
| *AN4034*-dR |  | GGAATCGAGACGCCTCCAG |  |
| *AN4034*-nested-F |  | CGCGCGATCCCTGCGCAGC |  |
| *AN4034*-nested-R |  | GCAATCTCAGCAATAACTC |  |
| *AN4034-*sgF |  | TAATACGACTCACTATAGGTGGAACAGGAACCTCAAT | T7 promoter + sgRNA |
| *AN4034-*sgR |  | TTCTAGCTCTAAAACATTGAGGTTCCTGTTCCACC |  |
| *pyrG*-F2 |  | GGATCTCAGAACAATATAC | *niaD* promoter |
| *niaD**.*P*-*F |  | CCATCGAGTCAGAGCCTAGCCAATGGCGGGCGCGGTGATTGAG |  |
| *niaD.*P*-*R |  | GAGTCTTGGGTATGGAGCC |  |
| *niaD*-nested-R |  | TGTGAGAGTATGGGATAGG | *pyrG-niaD*.P |
| ∆*gsdA*-uF | *gsdA* | GTGGTCAGCGTCGGAGGTG | ∆*gsdA* |
| ∆*gsdA*-uR |  | CAATATAGAAGCACTTACCTTCGCATCATCTTATCGGGCGGAATGTAG |  |
| ∆*gsdA*-dF |  | CCATCGAGTCAGAGCCTAGCCAAGGTTGTAGTTGTATAGGG |  |
| ∆*gsdA*-dR |  | CGATATTCAACAGATTTCG |  |
| ∆*gsdA*-nested-F |  | CTCCGTGGAGACGTTCTG |  |
| ∆*gsdA*-nested-R |  | CATGGACTCCGTCCGTCC |  |
| *AN2981*-sgF1 |  | TAATACGACTCACTATAGCCGAGGAGCAGCAGAATG | T7 promoter + sgRNA |
| *AN2981*-sgR1 |  | TTCTAGCTCTAAAACCCCATTCTGCTGCTCCTCG |  |
| *AN2981-*sgF2 |  | TAATACGACTCACTATAGACACCGTCATTGTCGTACT |  |
| *AN2981-*sgR2 |  | TTCTAGCTCTAAAACAGTACGACAATGACGGTGTC |  |
| *AN2981*-5′-F | *gsdA* | GGGTGAGCGAGAGCTCCATC | *nP*.*gsdA*, *nP*.*gsdA*/H_Flag, *nP*.*gsdA*/P_Gfp (*pyrG*-*niaD*.P-*gsdA* cassette) |
| *AN2981*-5′-R |  | TATAGAAGCACTTACCTTCGCATCCAGGGCCACGCCATGTCCGCGG |  |
| *AN2981*-3′-F |  | TTTCCTATCCCATACTCTCACAATGTCCGCCACGATAGCCCGCG |  |
| *AN2981*-3′-R |  | GAACTCGTTGCCAAAGCGC |  |
| *AN2981*-nested-F |  | GCCTCGGCGTCAGTTCTC |  |
| *AN2981*-nested-R |  | GGGCCTTCTGGAGATCACG |  |
| *gpdA.*P-F | g*pdA* | GCCTAGCCAAGTCAGACGGCGTAACCAAAAG | *gdpA* promoter |
| *gpdA.*P-R |  | TGTGATGTCTGCTCAAGCGG |  |
| *gpdA*-nested-R |  | CCGTTCATCTAAATGTCG |  |
| *AN2981*-*gpdA*.3′-F |  | CCCCGCTTGAGCAGACATCACAATGTCCGCCACGATAGCC | *gP*.*gsdA* (*pyrG*-*gpdA.*P-*gsdA* cassette) |
| *AN3954*-5′-F | *gndA* | CGTTTGTCGCGGATAACG | *gP*.*gndA* (*pyrG*-*gpdA.*P-*gndA* cassette) |
| *AN3954*-5′-R |  | ATAGAAGCACTTACCTTCGCATCTTGCTTAGAGACCCTGCCTAC |  |
| *AN3954*-3′-F |  | CCCCGCTTGAGCAGACATCACAATGACGGAAGAGTTGCTCCGTCAG |  |
| *AN3954*-3′-R |  | CTGCATGTCACCGTACTC |  |
| *AN3954*-nested.F |  | GAATCATCGGTGACCTCG |  |
| *AN3954*-nested.R |  | GACGTAGTGACCAGCACC |  |
| *prxA*-F | *prxA* | GAGCAGACATCACAATGTCTGGACTTAAGGCC | *gP*.*prxA* and *nP*.*gsdA*/*gP*.*prxA* |
| *prxA*-R |  | GGAGCTATTAAATCACTATTACAGGTGCTTGA |  |
| T*trpC*-F | *trpC* | GACTGTCATCAAGCACCTGTAATAGTGATTTAATAGCTCC |  |
| T*trpC*-R |  | GAAAGAAGGATTACCTCTAAAC |  |
| pUC19-*pyroA*-*gPprxA*-F | *Hind* III | GACCATGATTACGCCAAGCTTAGTCAGACGGCGTAACC |  |
| pUC19-*pyroA*-*gPprxA*-R | *Sma* I | CGAATTCGAGCTCGGTACCCGGGAAAGAAGGATTACCTC |  |
| **GFP-tagged PrxA** |  |  |  |
| *gfp*-cF |  | ATGAGTAAAGGAGAAGAAC | *gfp*-5GA |
| *gfp*-cR |  | GGCACCGGCTCCAGCGCCTGCACCAGCTCCTTTGTATAGTTCATCCATGC |  |
| *prxA*-uF | *prxA* | CATTCCCGGTGGCAAGCG |  |
| *prxA*-uR |  | GTGAAAAGTTCTTCTCCTTTACTCATTGTGTAAGATAGTGGTTGTTGG |  |
| *prxA*-dF |  | GTGCAGGCGCTGGAGCCGGTGCCATGTCTGGACTTAAGGCCGGTG |  |
| *prxA*-dR |  | GATACCTTTTCATAGCTTCG |  |
| pUC19-*prxA*-nested-F | *Sma* I | GGTCGACTCTAGAGGATCCCCGGGCTATTAAGGATACGTCTG |  |
| pUC19-*prxA*-nested-R | *Sma* I | CGAATTCGAGCTCGGTACCCGGGTGTATTGTCCTCGATC |  |
| **GFP-tagged NapA** |  |  |  |
| *gfp*-F |  | GGAGCTGGTGCAGGCGCTGGAGCCGGTGCCATGAGTAAAGGAGAAGAAC | 5GA-*gfp* |
| *gfp-R* |  | CGCGCACTTCTGCAGTTATTTGTATAGTTCATCC |  |
| *gfp-pyroA-F* |  | GGAGCTGGTGCAGGCGCTG | *gfp*::*pyroA* fragment |
| *gfp-pyroA-R* |  | GGATCCAGGAGTATACGGG |  |
| *napA-*5′-F |  | CTTTCGTGGGTGGGCTAGTC | *napA*-*gfp*-*pyroA* cassette |
| *napA-*5′-R |  | GCTCCAGCGCCTGCACCAGCTCCCACGCGGCCAATGATGTCATC |  |
| *napA-*3′ UTR-F |  | CAAAAACCCGTATACTCCTGGATCCTCGATTGTTCAATTCGATTCGC |  |
| *napA-*3′ UTR-R |  | CCTACAAACTCCATACTCAC |  |
| *napA-*5′-nested-F |  | GGTCTGGAACTTCCAGGTTG |  |
| *napA-*3′ UTR-nested-R |  | CTTCCCGTAGGCTTGTCC |  |
| **Flag-tagged HapC** |  |  |  |
| *hapC*-uF | *hapC* | CTGCTTGATGTTTGTTGCAG | H_Flag |
| *hapC*-dR |  | CCAGTATTGGTCTCATGC |  |
| *hapC*-Flag-R |  | CTTGTCGTCGTCGTCTTTGTAGTCATAAGATTCGCCACCAGCTCC |  |
| Flag-*hapC*-F |  | GACTACAAAGACGACGACGACAAGTAGTCTTTCTGTTACGG |  |
| pUC19-*pyroA*-*hapC*-uF | *Hind* III | GACCATGATTACGCCAAGCTTCAGGCCACAGACGAAGTG |  |
| pUC19-*pyroA*-*hapC*-dR | *Sma* I | CCGAATTCGAGCTCGGTACCCGGGCGAGACGCCTCCAGGAAG |  |
| **Recombinant** **AnG6PD** |  |  |  |
| pET28a-*gsdA*-F | *Xho* I | GTGGTGGTGGTGGTGCTCGAGATGTCCGCCACGATAG |  |
| pET28a-*gsdA*-R | *Nde* I | GTGCCGCGCGGCAGCCATATGCTACAGCCTGTTAG |  |
| *hapC*-check-F |  | GTCGTCGACCTCTCCCTC |  |
| *hapC*-check-R |  | CGGAGCTGGTGGCGAATC |  |
| *pyrG*-check-3′-F |  | CCGGAGTGTCTGAAGGTGC |  |
| *pyrG*-check-5′-R |  | CTTAGGGTACCGTCCACG |  |
| *argB*-check-3′-F |  | AGTCGTCCTAGCCAAGGTAG |  |
| *argB*-check-5′-R |  | AAGTGTCTTCGGAGTCAACC |  |
| *pyroA*-check-3′-F |  | GCTGGTGAGAACACATGC |  |
| *pyroA*-check-5′-R |  | CGCAGGGACTATATTGC |  |
| **q-RT-PCR** |  |  |  |
| q-RT-*actA-F* | *actA* | CGTCACCACTTTCAACTC | q-RT-PCR |
| q-RT-*actA-F* |  | GCGGTGATTTCCTTCTGC |  |
| q-RT-*prxA*.F | *prxA* | CCCCGCTGACGTTGTCTTC |  |
| q-RT-*prxA*.R |  | GAGGGCGAAGAGGATGACC |  |
| q-RT-*gsdA*.F | *gsdA* | GCAGCAGAATGGGAGCAC |  |
| q-RT-*gsdA*.R |  | GGGAAGGAACTTGTTGCG |  |
| q-RT-*gndA*.F | *gndA* | GCCGTATCATGCTCCTTG |  |
| q-RT-*gndA*.R |  | GTTGCTGTCGGGGAAGTG |  |
| q-RT-*napA*.F | *napA* | GCCTGACCAGCAGGACCTC |  |
| q-RT-*napA*.R |  | CCGTGGAAGCTTGACCTGG |  |

All the restriction enzymes sites in primer sequences were double underlined, and nucleotide sequence of Flag epitope were underline.

**
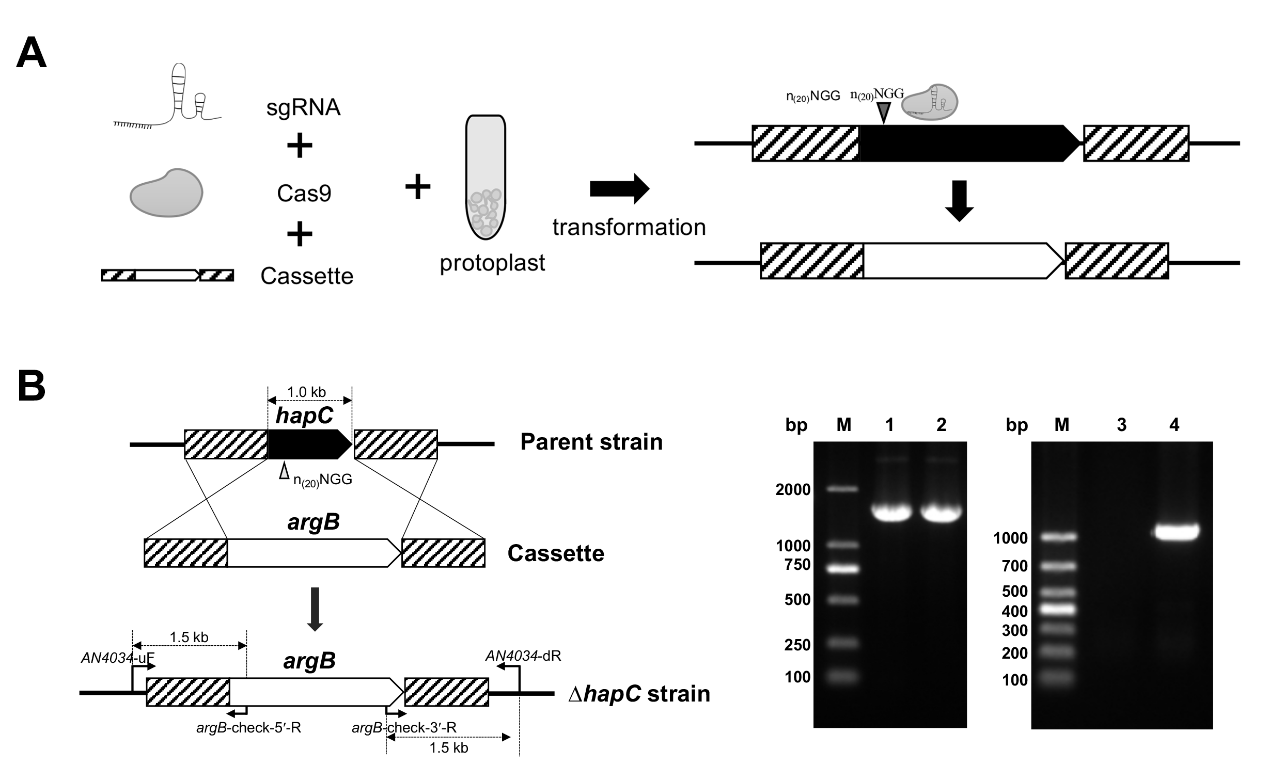
**

**Figure S1. CRISPR/Cas9-mediated disruptions of *hapC* gene in *A. nidulans***

**(A)** Schematic diagram of gene disruption by *in* *vitro* assembled Cas9/sgRNA and donor DNAs. **(B)** Gene replacement of the target gene loci via the homology-directed repair pathway. Left, Schematic diagram of locus changes of target genes before and after gene replacement. Right, Confirmation of gene disruptions by PCR using the indicated primer pairs. *hapC* disruptant was verified with primer pairs *AN4034*-uF/*pyrG*-check-5′-R (lane 1), *AN4034*-dR/*pyrG*-check-3′-R (lane 2), respectively; primer pair *hapC*-check-F/*hapC*-check-R was used to amplify AN4034 ORF in the parent (lane 3) and disruptant (lane 4) strains. M, marker. Primers are listed in Table S2.


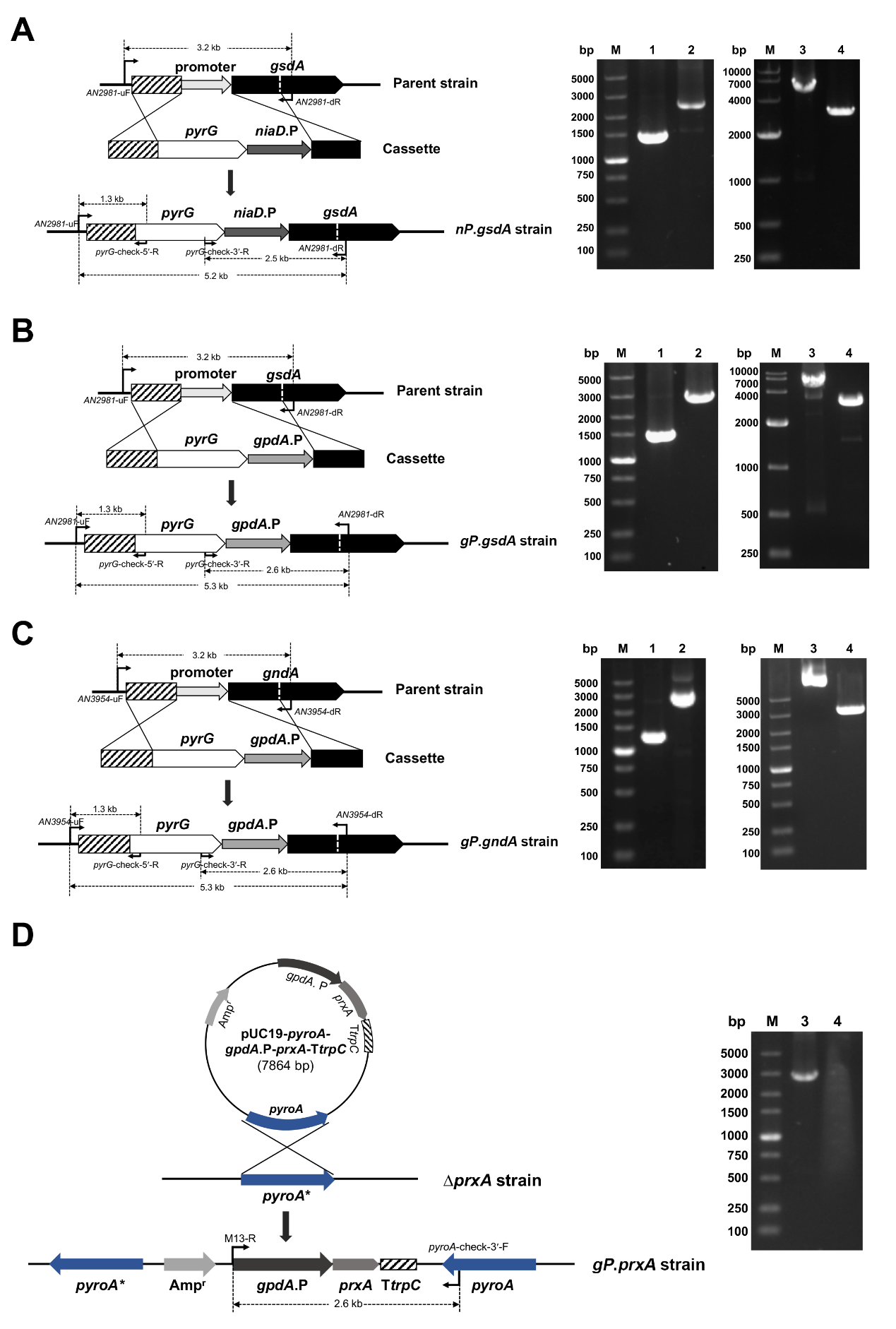


**Figure S2. Constructions of promoter substitution strains of *A. nidulans***

**(A-D)** Conditional promoter replacement strategy for homologous recombinant strains *nP*.*gsdA* (A), *gP*.*gsdA* (B), *gP*.*gndA* (C), *gP*.*prxA* (D) and validation of the corresponding recombinations by PCR with the indicated primers (right). The isolated *nP*.*gsdA* transformant was verified with primer pairs *AN2981*-uF/*pyrG*-check-5′-R (lane 1), *AN2981*-dR/*pyrG*-check-3′-R (lane 2), respectively; Primer pair *AN2981*-uF/*AN2981*-dR was used to amplify corresponding regions in the transformant (lane 3) and parent (lane 4) strains. M, marker. The similar methods were performed to validate the resultant transformants of *gP*.*gsdA* and *gP*.*gndA*. For verifying the indicated recombination in *gP*.*prxA*, primer pair M13-R/*pyroA*-check-3′-F was used to amplify corresponding regions in the transformant (lane 3) and parent (lane 4) strains. Primers are listed in Table S2.


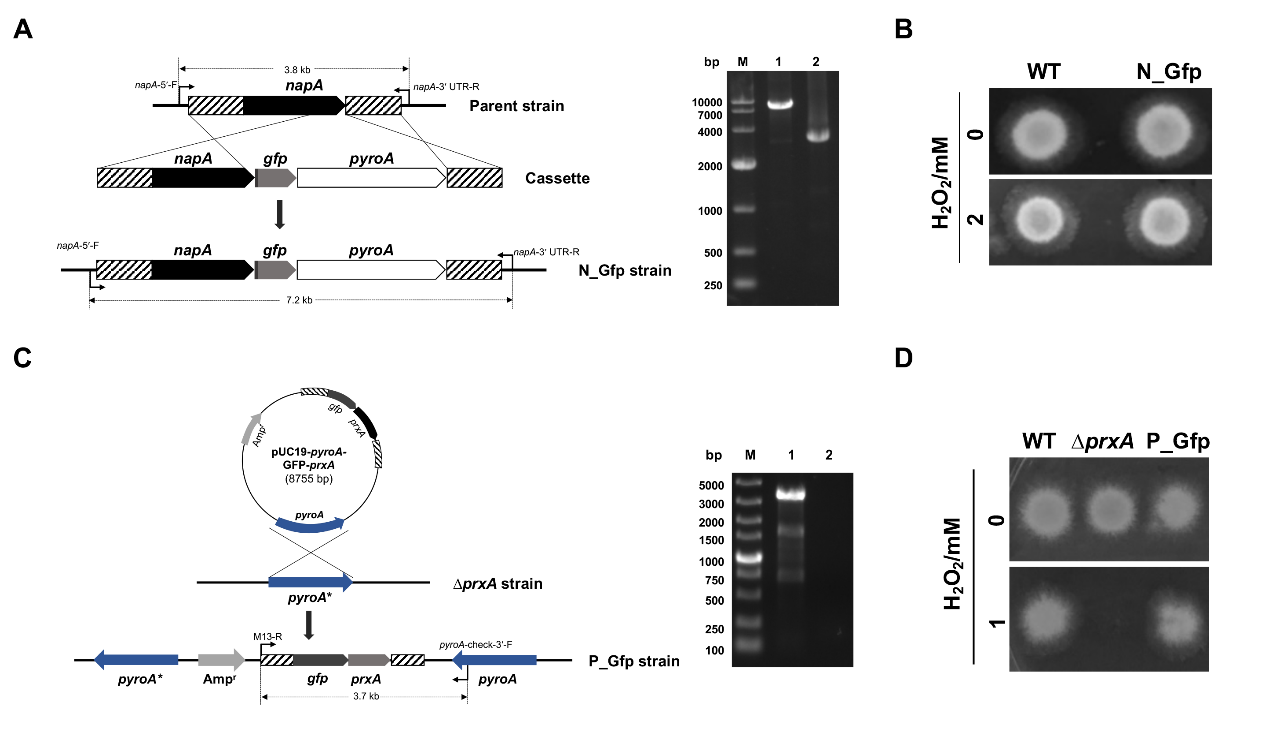


**Figure S3.** **Construction and phenotype analysis of GFP-tagged NapA strain and GFP-tagged PrxA strain**

**(A and C)** Schematic diagram of locus changes of *napA* gene (A) and *prxA* gene (C) before and after the gene replacements (left) and the corresponding confirmation using PCR (right). Primer pair *napA*-5′-F/*napA*-3′ UTR-R was used to amplify the corresponding region in the isolated transformant (lane 1) and the parent (lane 2) strains. M, marker. Primers are listed in Table S2. **(B)** GFP-tagged NapA strain (N_Gfp) shows similar H_2_O_2_ resistance to the control strain (WT_*argB*). Conidia (1 × 10^5^) from both strains were spotted on MM plates with or without 2 mM H_2_O_2_ and incubated at 37°C for 2 days. **(D)** GFP-tagged GFP strain (P_Gfp) recovered the H_2_O_2_ resistance and similar to the control strain (WT_*argB*). Conidia (1 × 10^5^) from both strains were spotted on MM plates with or without 1 mM H_2_O_2_ and incubated at 37°C for 2 days.


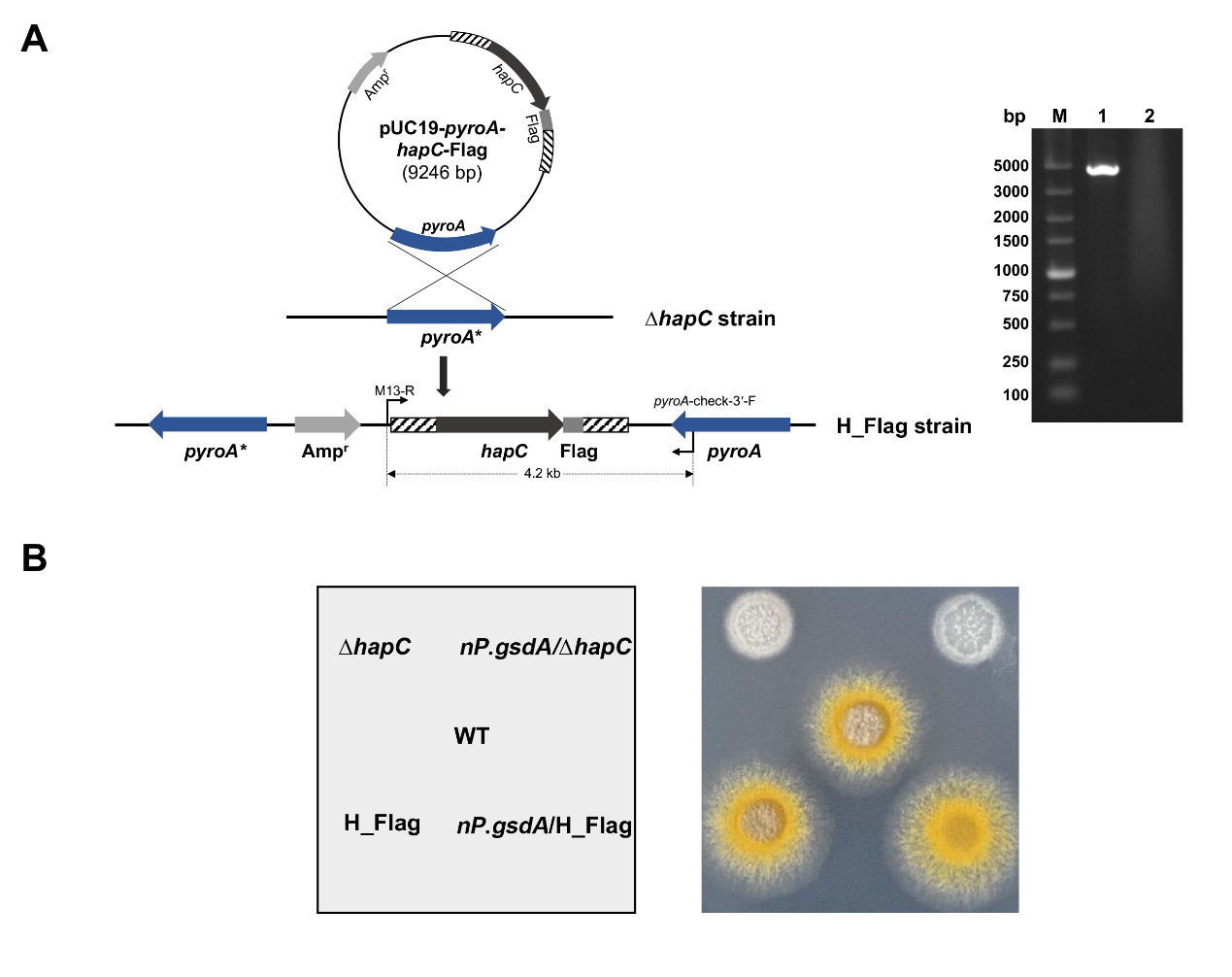


**Figure S4.** **Construction of Flag-tagged HapC strain and phenotype analysis of** **variants of *hapC* mutant under unstressed conditions**

**(A)** The recombinant plasmid pUC19-*pyroA*-*hapC*-Flag was used to transform to *∆hapC* to construct H_Flag strain. The indicated recombinant transformant was isolated and further confirmed by PCR with the primer pair M13-R/*pyroA*-check-3′-F (right, lane 1). Lane 2 showed the corresponding result of the control strain. Primer are listed in Table S2. **(B)** Conidia (1 × 10^5^) from the control (WT_*argB*), *∆hapC*, *nP*.*gsdA/∆hapC* and HapC::Flag fusion protein-expressing strains H_Flag, and *nP*.*gsdA*/H_Flag were spotted on MM plate without H_2_O_2_ and incubated at 37°C for 2 days.

**
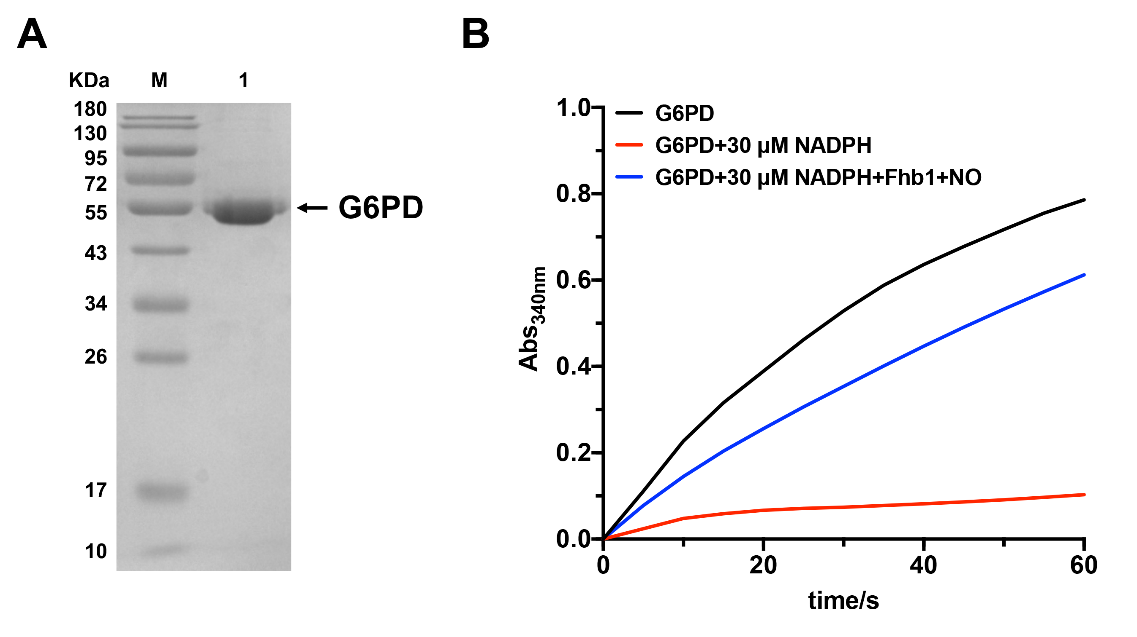
**

**Figure S5. Reversible** **inhibition of NADPH to G6PD activity**

**(A)** SDS-PAGE (12%) analysis of purified recombinant AnG6PD expressed by *E. coli*. M, maker. Lane 1, purified AnG6PD. **(B)** Activity of recombinant G6PD samples was estimated by generation of NADPH using a UV-Vis spectrophotometer at 340 nm. G6PD activity was measured in a 1 ml reaction mixture containing 5 mM glucose-6-phosphate, 0.3 mM NADP^+^, and 5 µM G6PD; 30 µM NADPH was added to the reaction mixture to evaluate the inhibition effects on G6PD activity. Further addition of 20 µM flavohemoglobin (Fhb1), and 50 µM NO donor MAHMA NONOate was used to consume NADPH for inhibition relief of G6PD activity.


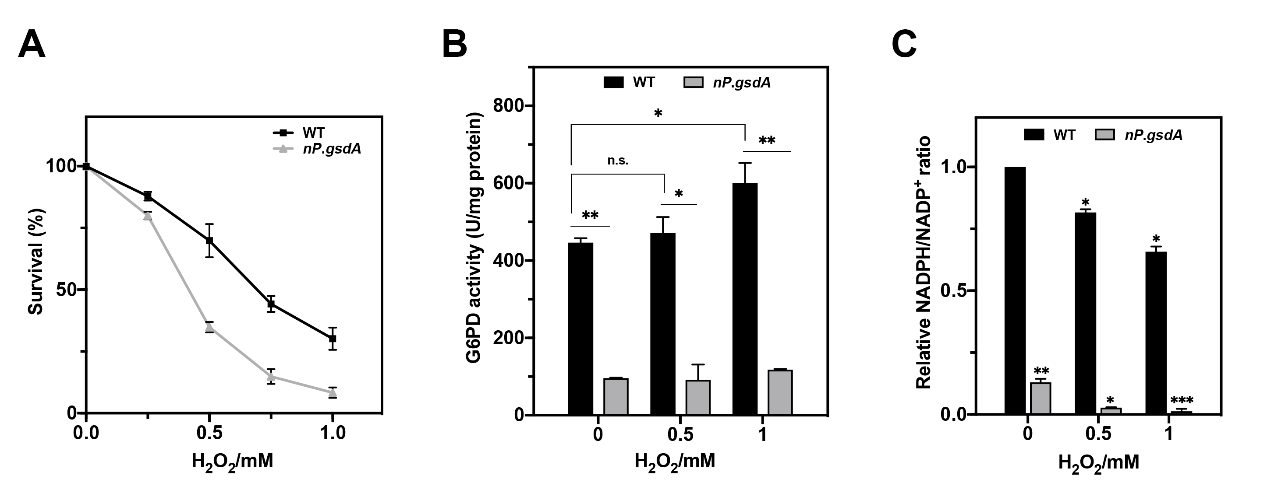


**Figure S6. Downregulation of *A. nidulans* *gsdA* is detrimental to fungal growth and oxidative stress resistance**

**(A)** Survival rates of WT (WT_*pyrG*) and *nP*.*gsdA* on MM plates using ammonium tartrate as nitrogen source under oxidative stress conditions. Fresh conidia (1 × 10^8^) of both strains were spread on MM plates containing the indicated concentrations of H_2_O_2_. Colonies were counted after a 48-h incubation, and survival rate are expressed as percentages of the CFU for strains incubated without H_2_O_2_. **(B-C)** G6PD activities and the relative NADPH/NADP^+^ ratio in WT and NH_4_^+^-repressed *nP*.*gsdA* strains before and after treatment of H_2_O_2_. Both strains were cultivated in MM liquid media using ammonium tartrate as the nitrogen source for 16 h, and then treated with the indicated concentrations of H_2_O_2_ for 30 min. (mean ± SD; n=3, **P <* 0.05, ***P* < 0.01, ****P* < 0.001; n.s*.*, not significant, one-way ANOVA.)

**
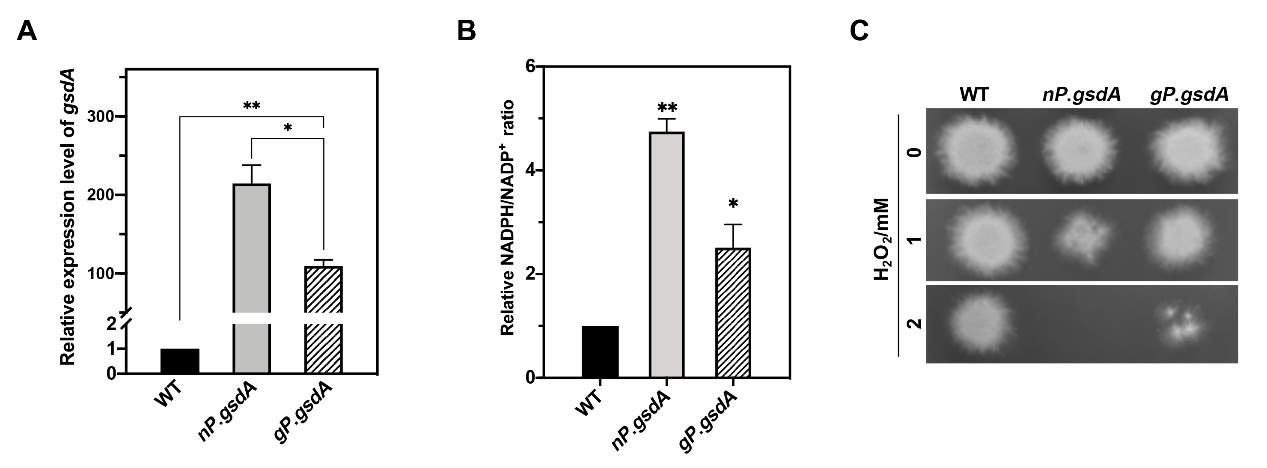
Figure S7. Replacement of *gsdA* native promoter with *gpdA* promoter also perturbed NADPH rhythm and impaired fungal resistance to H_2_O_2_**

**(A-B)** Relative expression levels of *gsdA* (A) and NADPH/NADP^+^ ratios (B) in WT (WT_*pyrG*), *nP*.*gsdA* and *gP*.*gsdA* (replacing *gsdA* promoter with *gpdA* promoter in WT) strains. All strains were precultivated in liquid NO_3_^−^-MM for 16 h and then exposed to 1 mM H_2_O_2_ for 30 min. The WT level of *gsdA* was set to 1, and the levels of *gsdA* in other strains were normalized to this. **(C)** Comparison of the H_2_O_2_ resistance of WT (WT_*pyrG*), *nP*.*gsdA* and *gP*.*gsdA* strains. (mean ± SD; n=3, **P <* 0.05, ***P* < 0.001, *t*-test.)


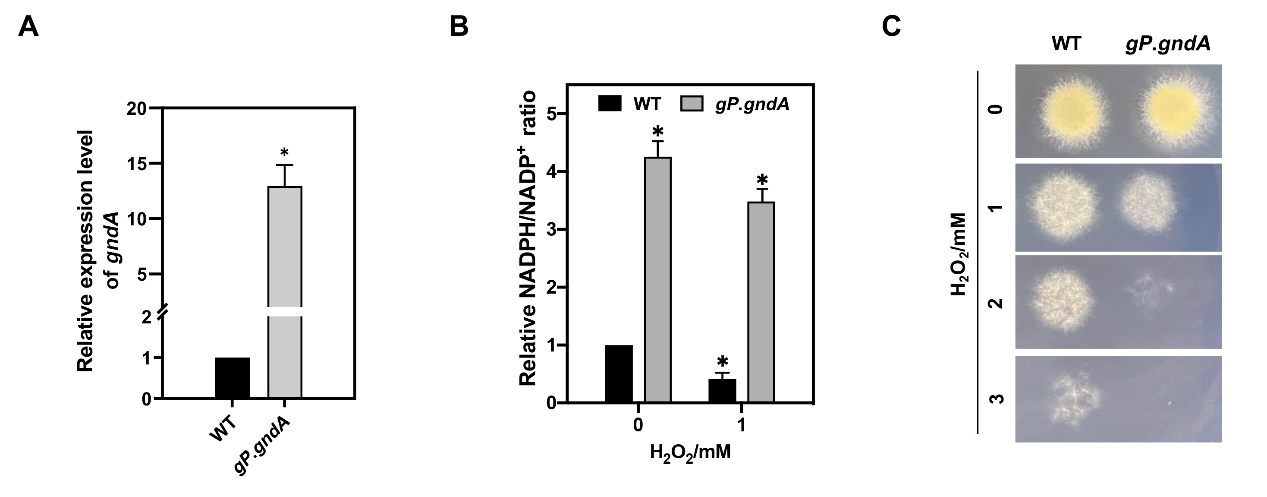


**Figure S8. Enhancing NADPH by overexpression of *gndA* impairs fungal resistance to H_2_O_2_**

**(A-B)** Relative expression levels of *gndA* (A) and NADPH/NADP^+^ ratios (B) in WT (WT_*pyrG*) and *gP*.*gndA* (replacing *gndA* promoter with *gpdA* promoter in WT) strains. All strains were precultivated in liquid NO_3_^−^-MM for 16 h and then exposed to 1 mM H_2_O_2_ for 30 min. The WT level of *gndA* was set to 1. **(C)** Comparison of the H_2_O_2_ resistance of WT (WT_*pyrG*) and *gP*.*gsdA* strains. (mean ± SD; n=3, **P <* 0.05, ***P* < 0.001, *t*-test.)


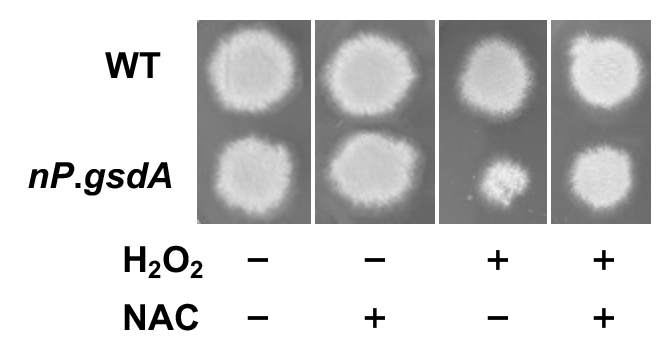


**Figure S9. Intracellular H_2_O_2_ accumulation accounts for the growth retardation of *nP.gsdA* strain under oxidative stress conditions**

Growth comparison of WT (WT_*pyrG*) and *nP*.*gsdA* strains under oxidative stresses or unstressed conditions. Conidia (1 × 10^5^) from both strains were spotted and cultivated for 2 days on NO_3_^−^-MM plates supplied with or without 10 mM NAC and 1 mM H_2_O_2_ as indicated by “+” and “−”, respectively.


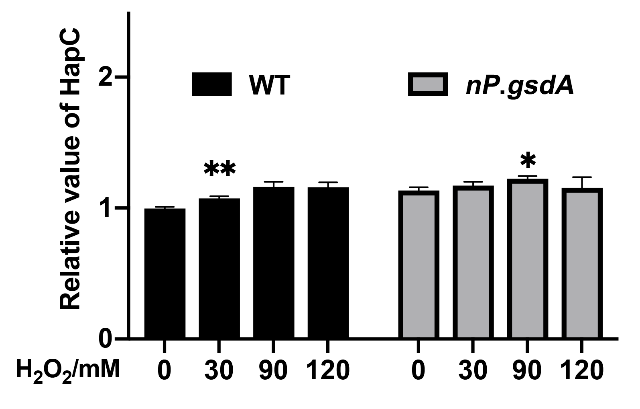


**Figure S10.** **Quantitative analysis of relative levels of intracellular HapC**

Total contents of intracellular HapC levels of WT and *nP*.*gsdA* strains at different periods are shown by the intensity of bands on denaturing and reducing PAGE. HapC levels were normalized to actin contents calculated by the same method for the further quantitative comparison. Each value represents the mean ± SD of mean of triplicate determinations. (mean ± SD; **P* < 0.05, ***P* < 0.01, one-way ANOVA.)
